# Tomato leaf curl Yunnan virus-encoded C4 induces cell division through enhancing stability of Cyclin D 1.1 via impairing NbSKη -mediated phosphorylation in *Nicotiana benthamiana*

**DOI:** 10.1371/journal.ppat.1006789

**Published:** 2018-01-02

**Authors:** Yuzhen Mei, Xiuling Yang, Changjun Huang, Xiuren Zhang, Xueping Zhou

**Affiliations:** 1 State Key Laboratory of Rice Biology, Institute of Biotechnology, Zhejiang University, Hangzhou, Zhejiang, China; 2 State Key Laboratory for Biology of Plant Diseases and Insect Pests, Institute of Plant Protection, Chinese Academy of Agricultural Sciences, Beijing, China; 3 Department of Biochemistry & Biophysics, Texas A&M University, College Station, United States of America; 4 Institute for Plant Genomics and Biotechnology, Texas A&M University, College Station, United States of America; Universidade Federal de Viçosa, BRAZIL

## Abstract

The whitefly-transmitted geminiviruses induce severe developmental abnormalities in plants. Geminivirus-encoded C4 protein functions as one of viral symptom determinants that could induce abnormal cell division. However, the molecular mechanism by which C4 contributes to cell division induction remains unclear. Here we report that tomato leaf curl Yunnan virus (TLCYnV) C4 interacts with a glycogen synthase kinase 3 (GSK3)/SHAGGY-like kinase, designed NbSKη, in *Nicotiana benthamiana*. Pro32, Asn34 and Thr35 of TLCYnV C4 are critical for its interaction with NbSKη and required for C4-induced typical symptoms. Interestingly, TLCYnV C4 directs NbSKη to the membrane and reduces the nuclear-accumulation of NbSKη. The relocalization of NbSKη impairs phosphorylation dependent degradation on its substrate-Cyclin D1.1 (NbCycD1;1), thereby increasing the accumulation level of NbCycD1;1 and inducing the cell division. Moreover, *NbSKη-RNAi*, *35S*::*NbCycD1;1* transgenic *N*. *benthamiana* plants have the similar phenotype as *35S*::*C4* transgenic *N*. *benthamiana* plants on callus-like tissue formation resulted from abnormal cell division induction. Thus, this study provides new insights into mechanism of how a viral protein hijacks NbSKη to induce abnormal cell division in plants.

## Author summary

Induction of cell division is essential for DNA virus replication in differentiated plant cells. To achieve efficient replication, DNA viruses encode C4 protein to induce cell division. However, the mechanism of C4-induced cell division is still largely unknown. This study demonstrates that tomato leaf curl Yunnan virus (TLCYnV) C4 protein hijacks NbSKη, directs NbSKη to the membrane and reduces the nuclear-accumulation level of NbSKη, which protects its substrate-Cyclin D1.1 (NbCycD1;1) from phosphorylation by NbSKη and proteasomal degradation in the nucleus, thereby increasing the nuclear-accumulation level of NbCycD1;1 and inducing the cell division.

## Introduction

Geminiviruses cause devastating diseases in fields and economic losses in agriculture in the world [1–7]. They have a single-stranded DNA genome, 2.5~3.0 kb, encoding 6~7 genes. Some of the viruses are associated with satellites [5]. Many geminiviruses replicate in differentiated cells that no longer contain detectable levels of host DNA polymerases and associated factors. To overcome this barrier and make a suitable environment for replication, geminiviruses induce the accumulation of DNA replication machinery in mature plant cells [8]. Maize streak virus (MSV) RepA protein stimulates cell division and calli growth of maize cultures through binding to the retinoblastoma-related (RBR) protein [9]. Beet severe curly top virus (BSCTV) C4 protein induces the expression of *RKP*, a gene encodes a RING finger E3 ligase, to regulate cell cycle [10].

Tomato leaf curl Yunnan virus (TLCYnV) is thought as a recombinant virus derived from two geminivirus species: tomato yellow leaf curl China virus (TYLCCNV) as major parent and pepper yellow leaf curl China virus as the donor of the *C4* gene and the partial intergenic region (IR) [11]. Geminiviral C4 has been often known as one of viral symptom determinants and has an effect on cell division induction [12–16]. It has been reported that geminiviral C4 regulates many genes, or interacts with a few host factors to induce hyperplasia symptoms, including *Arabidopsis thaliana* shaggy-related protein kinases AtSKη and AtSKζ[12, 17].

AtSKη is a homologue of mammalian glycogen synthase kinase 3 (GSK3). GSK3 plays a key role in glycogen metabolism [18, 19]. GSK3 is also involved in numerous signal transduction pathways [20, 21], including insulin signaling [22], and cell fate specification during embryonic development [23]. There are two GSK3 isoforms in mammals that encoded by two genes, *GSK3α* and *GSK3β*. The two isoforms have a conserved middle kinase domain, but divergent N- and C-terminus [24]. In *Arabidopsis thaliana*, *SKη* is a member of gene family, *AtSKs* [25]. AtSKη is a close othorlog of animal GSK3β and their catalytic domains share 70% sequence identity [26].

*AtSKη*, also known as *BIN2* (*Brassinosteroid-insensitive 2*), is a negative regulator in the brassinosteroid (BR) signal transduction pathway [26]. BRs are steroid hormones that are widely distributed in plant kingdom, and play regulatory roles in plant growth and development [27, 28]. Briefly, AtSKη/BIN2 functions through phosphorylation of downstream transcription factors to regulate BR signaling pathway, such as BRASSINAZOLE-RESISTANT 1 (BZR1) [29, 30] and BRI1-EMS SUPPRESSOR 1 (BES1, also named as BZR2) [31, 32]. In the presence of BR, membrane-localized receptor BRASSINODTEROID INSENSITIVE 1 (BRI1) is elicited when BR binds to the extracellular domain of BRI1 receptor. Once the signaling cascade is induced, BRI1 SUPPRESSOR 1 (BSU1) phosphatase dephosphorylates the AtSKη/BIN2 kinase on Tyr-200 to inactivate AtSKη/BIN2 in cytoplasm [33]. The unphosphorylated form of BZR1 and BES1 could bind the promoter of BR-regulated target genes, activating the expression of downstream BR-responding genes or repressing the expression of BR biosynthesis genes, such as *CONSTITUTIVE PHOTOMORPHOGENESIS AND DWARFISM* (*CPD*) and *DWARF4* (*DWF4*) when BR signaling is enough to maintain normal plant growth and development. In the absence of BR, BZR1 and BES1 could be inactivated by AtSKη/BIN2-mediated phosphorylation in the nucleus, and inactivated BZR1 and BES1 could not induce the BR-regulated gene expression [34].

BRs also have effects on cell division [35–38]. One model has been proposed that BRs could enhance the expression level of Cyclin D3 in *Arabidopsis* callus and suspension cells to promote cell division [35]. Notably, the mechanism of the promotion of cell division by brassinolide (BL) treatment is distinct from that regulated by the balance of auxin and cytokinin [36]. Cyclin D acts as a growth sensor that integrates environment signals with the cell cycle machinery [39]. Constitutive expression of CYCD3;1 could produce calli in leaf explants [40]. *35S*::*CYCD3;1* transgenic *A*. *thaliana* plants results in hyperplasia of leaf epidermal tissue caused by hyperproliferation of leaf cells [41]. Expression of CycD1;1 in G0 cells accelerates entry into S-phase and advances mitotic entry, CycD1;1 could promote G0/G1/S progression [42]. However, the molecular mechanism of how AtSKη/BIN2 regulates Cyclin D-related cell cycle and how geminivirus C4 proteins interfere AtSKη/BIN2 to induce cell division remains largely unknown.

Here, we validated the interaction between TLCYnV C4 and NbSKη. Moreover, we pinpointed that Pro32, Asn34 and Thr35 of TLCYnV C4 were critical for its interaction with NbSKη. NbSKη-interaction compromised C4 mutants reduced the severity of C4-induced symptoms in a potato virus X (PVX)-based expression system. We found that TLCYnV C4 directs NbSKη to the membrane through interaction and reduces the nuclear accumulation level of NbSKη. The redistribution of NbSKη by C4 contributes to re-activate the abnormal cell division in mature leaves, whereas NbSKη-interaction compromised C4 mutants could not do so. Notably, we found that NbSKη negatively regulates cell cycle by phosphorylation of NbCycD1;1, thus triggering its degradation through 26S proteasome. However, C4 protein could override this progress by blocking NbSKη activity and inhibiting degradation of NbCycD1;1. These results suggest that TLCYnV C4 reactivates the cell cycle in differentiated cells by changing the localization of NbSKη in *N*. *benthamiana*.

## Results

### The interaction between TLCYnV C4 and NbSKη is critical for symptom development

To elucidate the molecular mechanism of how geminivirus manipulates plant cell cycle to favour its replication, we screened a *N*. *benthamiana* cDNA library by a yeast two-hybrid (Y2H) to identify C4-targeted host factors. Among the C4-interacting clones, two overlapping cDNAs encoding a GSK3/SHAGGY-like kinase were recovered. Interestingly, the kinase was an *Arabidopsis* homolog previously identified as the interactor of C4 by independent groups [12, 16]. We named this gene as *NbSKη* which encodes 383 amino acids (aa) that share 89% identity with that of AtSKη/BIN2. We validated the interaction of TLCYnV C4 and NbSKη in Y2H and bimolecular fluorescence complementation (BiFC) assays (S1A and S1B Fig).

A phylogenetic analysis revealed that NbSKη has a close homology with NsSKη in *N*. *sylvestris*, SlSKη in *Solanum lycopersicum*, BIN2-like protein PSK9 in *Petunia hybrid*, VvSKη in *Vitis vinifera*, GaSKη in *Gossypium arboretum*, and AtSKη in *A*. *thaliana* (S1C Fig). A computational analysis through SMART program (SMART, http://smart.embl-heidelberg.de) predicted NbSKη to contain a catalytic domain of serine or threonine-specific kinases (35–327 aa) (S1D Fig).

**Fig 1:**
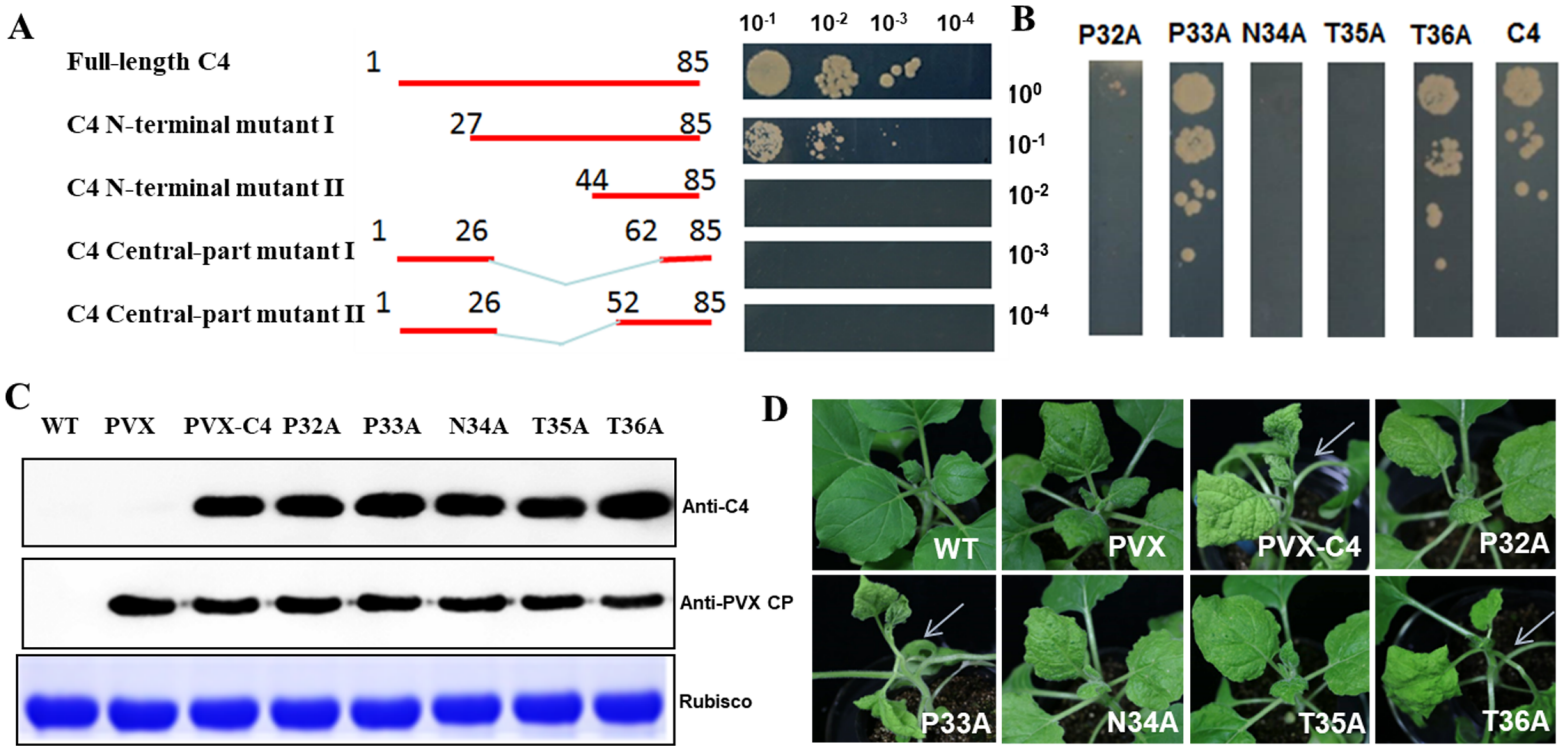
Key sites of TLCYnV C4 vital for its interaction with NbSKη. **(A)** Schematic representation of the truncated mutants of TLCYnV C4 and the C4 mini-domain associated with the interaction. Yeast strain Gold co-transformed with the indicated plasmids were subjected to 10-fold serial dilutions, and grown on a SD/-Leu/-Trp/-His/-Ade medium. **(B)** Key points of TLCYnV C4 important for the interaction. Yeast strain Gold co-transformed with the indicated plasmids were subjected to 10-fold serial dilutions, and grown on a SD/-Leu/-Trp/-His/-Ade medium. **(C)** Western blot analysis of C4 wild-type and mutant proteins in systemic leaves of the infected plants. C4 and PVX CP proteins were detected using TLCYnV C4 and PVX-CP specific polyclonal antibodies. Rubisco was the loading control. **(D)** Symptoms in *N*. *benthamiana* plants infected with PVX-based vector harboring C4 or C4 mutant (P32A, P33A, N34A, T35A or T36A). Photographs were taken at 8 dpi. Arrowheads indicate the symptoms induced by PVX-C4.

To delineate the interaction interface of C4-NbSKη interaction, we initially generated four C4 deletion mutants and repeated the Y2H assays. We found that a 17 amino acids polypeptide in C4 middle-part (27–43 aa) was crucial for the interaction (Fig 1A). Next, we carried out site-directed mutagenesis to pinpoint the key residue(s) involved in the interaction. Y2H and BiFC assays showed that P32, N34 and T35 of TLCYnV C4 were vital for the interaction since each single point mutation led to loss of its interaction (Fig 1B and S2 Fig).

To study whether the mutations identified above have biological relevance, we expressed C4 and its variants in *N*. *benthamiana* plants using a PVX-based vector. Western blot assays showed that the accumulation levels of C4 and C4 variants in systemic leaves were similar in plant at 8 days post-infiltration (dpi) (Fig 1C). Notably, expression of wild-type *C4* gene or the *C4* mutants (P33A or T36A) that maintain the interaction with NbSKη produced severe chlorotic spots, and foliar distortion, consisting with previous reports about C4 as a viral symptom determinant. As expected, expression of NbSKη-interaction compromised *C4* mutants (P32A, N34A or T35A) only caused PVX-like symptoms with mild chlorotic spots on leaves, and thus had no significant influence on plant development (Fig 1D). Also, TLCYnV infectious clone harboring C4 mutants deficient of NbSKη binding could not infect *N*. *benthamiana* systemically (S3 Fig). These results suggest that P32, N34 and T35 of TLCYnV C4 are essential for C4-NbSKη interaction and viral symptom development.

### TLCYnV C4 is a membrane-localized protein and directs NbSKη to cytoplasmic membrane when co-expressed

**Fig 2:**
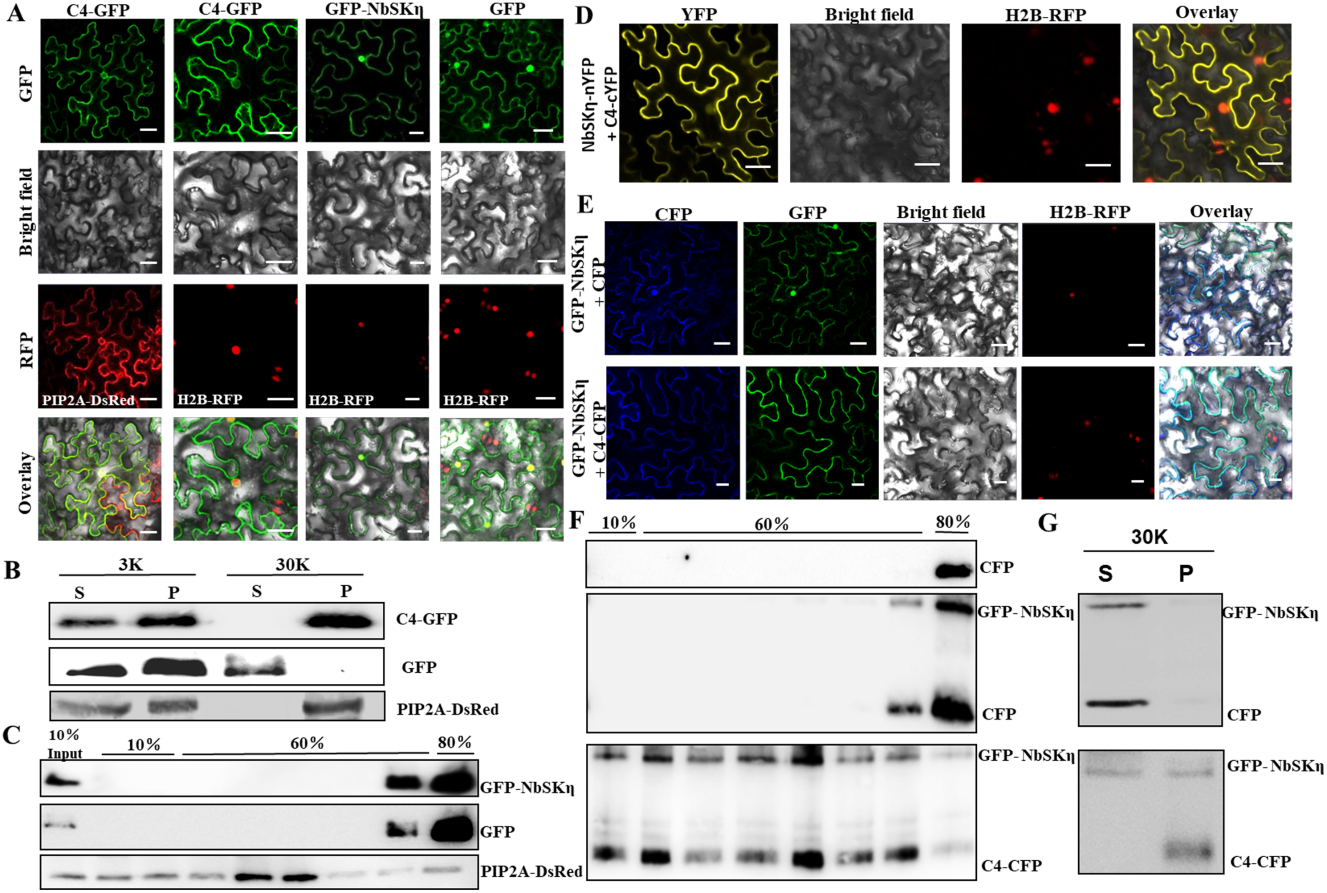
TLCYnV directs NbSKη to cytoplasmic membrane. **(A)** Subcellular localization of C4-GFP or GFP-NbSKη fusion protein in *N*. *benthamiana* epidermal cells. GFP-tagged proteins were expressed in the transgenic *N*. *benthamiana* plants expressing the nucleus marker H2B-RFP (2–4 rows), or in the *N*. *benthamiana* plants expressing the cytoplasmic membrane marker (first row). Scale bar = 50 μm. **(B)** Subcellular fractionation of C4-GFP, GFP, and PIP2A-DsRed. Plant tissues expressing C4-GFP, GFP, or PIP2A-DsRed were fractionated into soluble (S) and membrane-enriched (P) fractions. GFP and PIP2A-DsRed acted as the markers of soluble fraction and membrane-enriched fraction, respectively. 3K, the extracts following centrifugation at 3,000 *g*; 30K, the extracts following centrifugation at 30,000 *g*. **(C)** Membrane flotation assays of GFP-NbSKη, GFP, and PIP2A-DsRed. GFP-NbSKη and PIP2A-DsRed were detected using monoclonal antibodies specifically against GFP and RFP, respectively. **(D)** BiFC analysis of C4/NbSKη interaction position in epidermal cells of H2B-RFP transgenic *N*. *benthamiana* plants. Scale bar = 50 μm. **(E)** Distribution patterns of GFP-NbSKη in the presence of CFP or C4-CFP expressed in the H2B-RFP transgenic *N*. *benthamiana* epidermal cells. Scale bar = 50 μm. **(F)** Membrane flotation assays of plant extracts expressing CFP alone or co-expressing GFP-NbSKη with CFP or C4-CFP. CFP, C4-CFP, and GFP-NbSKη were detected using a rabbit monoclonal antibody raised against GFP. **(G)** Subcellular fractionation of plant tissues prepared from *N*. *benthamiana* plants expressing GFP-NbSKη with CFP or C4-CFP.

To investigate the subcellular localization of TLCYnV C4, C4-GFP (intact GFP fused to the C-terminus of C4) and plasma membrane intrinsic protein 2A (PIP2A)-DsRed (intact DsRed fused to the C-terminus of PIP2A) under the control of cauliflower mosaic virus (CaMV) *35S* promoter were co-infiltrated into *N*. *benthamiana*. PIP2A protein acts as a marker for membrane-localization [43]. GFP and DsRed fluorescences in local inoculated leaves were observed by confocal microscope at 48 hours post infiltration (hpi). Confocal microscope assays showed that C4-GFP was coincided with PIP2A-DsRed, whereas only residual signal of C4-GFP was detected in the nucleus (Fig 2A). This result indicates that C4-GFP was predominantly localized to plasma membrane and endo-membrane system. Such observation was further supported by expression of C4-GFP in transgenic *N*. *benthamiana* lines which expressed histone H2B-Red Fluorescent Protein fusion (H2B-RFP) protein. Major membrane localization pattern could be also observed for C4-GFP (Fig 2A). We next conducted subcellular fractionation assays. Protein extracts were prepared from plant tissues expressing GFP, C4-GFP and PIP2A-DsRed, respectively, and separated by high-speed centrifugation into pellet (P30) and supernatant (S30) fractions. Again, western blot analysis indicated that C4-GFP but not GFP alone was only present in the P30 fraction, similar as that of PIP2A-DsRed which acts as a membrane-localization marker (Fig 2B). Thus, this result, together with the confocal experimental assays, shows that TLCYnV C4 is a membrane-localized protein.

In parallel, we examined the cellular distribution of NbSKη protein. GFP-NbSKη was transiently expressed in H2B-RFP transgenic *N*. *benthamiana* leaf epidermal cells by agro-infiltration. Confocal fluorescence imaging showed that GFP-NbSKη was mainly localized in the nucleus, and also widely diffused in the cytosol (Fig 2A). To determine whether NbSKη is trafficked to the membrane, membrane-flotation assays were performed by using sucrose density gradient centrifugation. Identical to the soluble protein GFP, but in contrast to the membrane-localized PIP2A-DsRed, GFP-NbSKη was only detected in the 80% sucrose fraction (Fig 2C). These results further indicate that NbSKη is a soluble protein in the cytosol and nucleus.

To identify the subcellular interaction sites between NbSKη and C4, BiFC assays were performed by using C4-cYFP [C4 fused to the N-terminus of YFP C-terminal fragment (159–238 aa)] and NbSKη-nYFP [NbSKη fused to the N-terminus of YFP N-terminal fragment (1–158 aa)]. Confocal fluorescence imaging shows that the interaction signal was predominantly detected on the cyto-membrane but barely in the nucleus (Fig 2D). The fluorescence distribution pattern of NbSKη and C4 interaction signal was similar as that of C4-GFP alone but not as that of GFP-NbSKη. This result suggests that co-expression of TLCYnV C4 may alter the distribution pattern of GFP-NbSKη.

To further test this hypothesis, C4-CFP (cyan fluorescent protein fused to the C-terminus of C4) was co-expressed with GFP-NbSKη in epidermal cells of H2B-RFP transgenic plants. When GFP-NbSKη was co-expressed with C4-CFP, less GFP-NbSKη signal remained in the nucleus compared to that expressing GFP-NbSKη alone (Fig 2E). These results indicated that C4 indeed decreased the portion of the nuclear-localized NbSKη, and increased the amount of membrane-associated NbSKη. We next repeated membrane flotation assays using plant tissues co-expressing GFP-NbSKη with CFP or C4-CFP. Fig 2F showed that different from the combination of CFP and GFP-NbSKη, co-expression of C4-CFP and GFP-NbSKη shifted the protein distribution from the bottom sucrose gradient (80%) to top ones even like in the 10% fraction. Further, we performed subcellular fractionation assays and observed that GFP-NbSKη and CFP were present in the S30 fraction. However, when GFP-NbSKη was co-expressed with C4-CFP, some of GFP-NbSKη appeared in the P30 fraction (Fig 2G). These results indicate that the expression of C4-CFP directs GFP-NbSKη to the cyto-membrane.

To further test whether C4 decreased the accumulation level of nuclear-localized NbSKη, we performed the nuclear-cytoplasmic fractionation assays by using wild-type and C4 transgenic *N*. *benthamiana* plants. We used histone H3 and phosphoenolpyruvate carboxylase (PEPC) as the nuclear- or cytoplasmic-localized proteins, respectively. S4 Fig showed that the accumulation level of nuclear-localized NbSKη in C4-transgenic *N*. *benthamiana* plants was lower than that in wild-type *N*. *benthamiana* plants.

**Fig 3:**
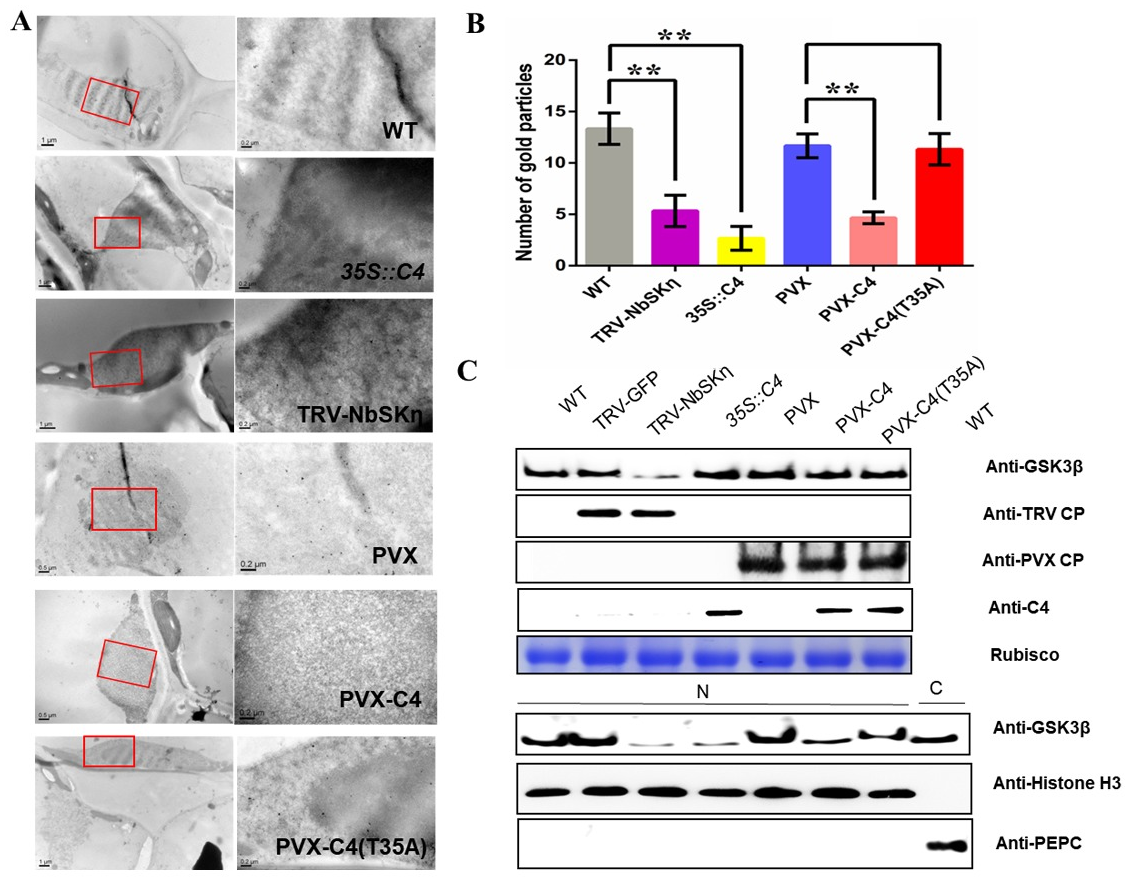
Immuno-cytochemistry and immunoblot analysis of the nuclear-localized accumulation of NbSKη under different treatments. **(A)** Analysis of nuclear-localized NbSKη accumulation through immuno-cytochemistry and electron microscopy. Thin sections were prepared from *N*. *benthamiana* leaf tissues under different treatments. All the sections were probed with anti-GSK3β polyclonal antibody followed by a protein A-gold conjugate. Scale bar = 0.2 μm. **(B)** Average number of gold particles per 0.25 um^2^ in three randomly selected nuclei in sections from various treatments. ** represents significant difference (P value <0.01) between treatments tested by *t-*test. **(C)** Nuclear-cytoplasmic fractionation analysis of the accumulation of the nuclear-localized NbSKη in plant tissues under different treatments. Western blot analysis was conducted with the antibodies specific to the indicated proteins.

Finally, to obtain the direct evidence for C4-mediated relocalization of NbSKη, we prepared thin sections from the fixed and embedded *N*. *benthamiana* leaf tissues. Because the commercially-available anti-GSK3β antibody could specially recognize NbSKη (S5 Fig), we analyzed NbSKη localization in the fixed section using an anti-GSK3β antibody followed by a protein A-gold conjugate. Under the electron microscope, the average number of gold particles in the nuclei of C4 transgenic, *NbSKη*-silenced and PVX-C4-infected cells was significantly less than that of the wild type, PVX- and PVX-C4(T35A)-infected cells (Fig 3A and 3B). To validate the electron microscopy results, we performed the nuclear-cytoplasmic fractionation assays to detect the accumulation level of NbSKη in the nucleus. The accumulation levels of NbSKη in the nucleus in C4 transgenic plants, NbSK*η*-silenced and PVX-C4-infected plants were lower than that in wild type, tobacco rattle virus (TRV) vector control- (TRV-GFP-), PVX-, and PVX-C4(T35A)-infected plants (Fig 3C).

To investigate whether TLCYnV C4-NbSKη interaction is necessary for the re-location of NbSKη in the presence of C4, we repeated the confocal assays with various C4 mutants in the H2B-RFP transgenic *N*. *benthamiana*. Interestingly, similar to wild-type C4, P33A or T36A mutants clearly relocalized the distribution of NbSKη. In contrast, NbSKη-interaction compromised TLCYnV C4 mutants (P32A, N34A or T35A) had no obvious impact on the relocalization of NbSKη (S6A and S6B Fig). These results were further confirmed by nuclear-cytoplasmic fractionation assays. Western blot results indicated that the nuclear accumulation level of NbSKη protein was significantly lower in plants inoculated with PVX-C4, PVX-C4 (P33A) or PVX-C4(T36A) than that in plants infected with PVX harboring C4-NbSKη interaction compromised mutants (S6C Fig). All together, the results indicate that the interaction between C4 and NbSKη decreases the nuclear-localization portion and increases the membrane-localized NbSKη in *N*. *benthamiana*.

### *NbSKη*-silenced tobacco plants phenocopies PVX-C4-infected tobacco plants

**Fig 4:**
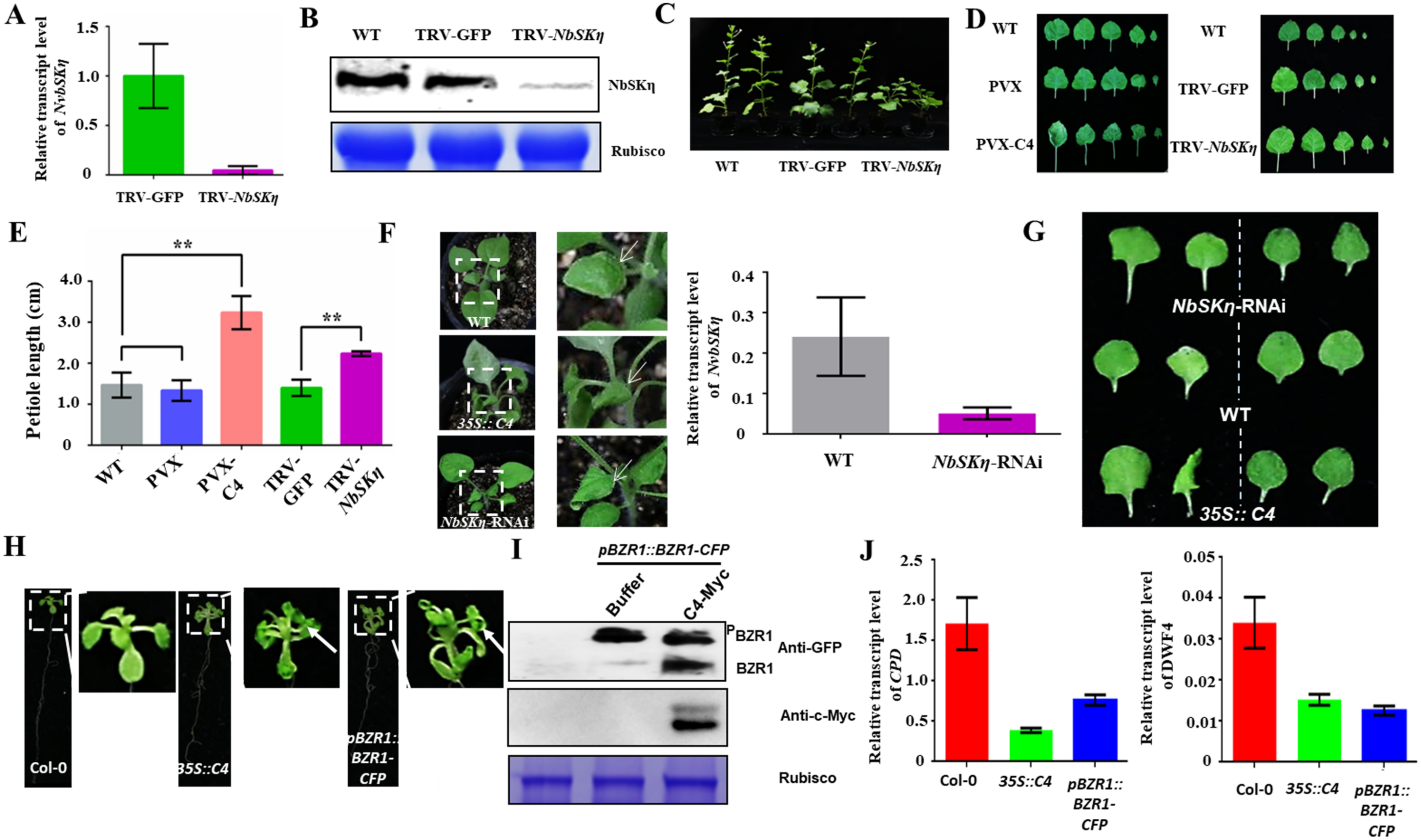
*NbSKη-*silenced *N*. *benthamiana* plants display the phenotype similar to *N*. *benthamiana* plants infected by PVX-C4. **(A** and **B)** qRT-PCR (A) and western blot (B) assays of *NbSKη* gene expression in wild-type (WT) and *NbSKη*-silenced plants. Relative accumulation level of *NbSKη* transcripts is normalized against the amount of *actin* transcript. Error bar denotes the standard deviation of three biological replicates. **(C)** Growth of the wild-type (WT), mock (TRV-GFP) and *NbSKη*-silenced (TRV-*NbSKη*) *N*. *benthamiana* plants at 35 dpi. **(D)** The leaf petioles of *NbSKη-*silenced *N*. *benthamiana* leave mimics that of *N*. *benthamiana* leave inoculated with PVX-C4. The phenotype of 1st-5th leaves of plants under different treatments was shown. **(E)** The petiole length of the 3rd leaf of plants under different treatments. Each treatment had fifteen plants at the same developmental stage. ** represent significant difference (P value <0.01) between treatments tested by *t*-test. **(F)** The phenotype of WT, *35S*::*C4* or *NbSKη*-RNAi transgenic *N*. *benthamiana* plants (left) and qRT-PCR analysis of *NbSKη* gene expression in WT and *NbSKη*-RNAi transgenic plants (right). Relative accumulation level of *NbSKη* transcripts is normalized against the amount of *actin* transcript. Error bar denotes the standard deviation of three biological replicates. **(G)** The petiole length of the 2nd (right column) and 3rd (left column) leaves of WT, *35S*::*C4* transgenic or *NbSKη*-RNAi transgenic *N*. *benthamiana* plants. **(H)** Images of Col-0, *pBZR1*::*BZR-CFP* (BZR1 overexpression *A*. *thaliana*) or *35S*::*TLCYnV C4* transgenic *A*. *thaliana*. Arrowheads indicate the downward leaf curling of the leaves. **(I)** Western blot analysis of BZR1-CFP phosphorylation level. The presence of TLCYnV C4 could decrease the level of phosphorylated BZR1-CFP. BZR1-CFP was detected using a monoclonal antibody against GFP. C4-Myc was detected using a monoclonal antibody against Myc. BZR1-CFP and phosphorylated BZR1-CFP were designated as BZR1 and ^P^BZR1, respectively. **(J)** Quantitative PCR analysis of relative expression levels of BZR1-targeted genes (*CPD* and *DWF4*) in Col-0, *35S*::*C4* transgenic or *pBZR1*::*BZR1-CFP* transgenic *A*. *thaliana*.

To investigate whether NbSKη contributes to C4-induced symptoms, we silenced *NbSKη* gene in tobacco plants using a TRV-based vector. Real-time quantitative PCR (qRT-PCR) analysis revealed that the transcripts of *NbSKη* were significantly reduced (over 75%) in *NbSKη-*silenced plants compared with the amount in mock plants inoculated with vector control (TRV-GFP) (Fig 4A). Western blot analysis also showed that the protein level of NbSKη in *NbSKη-*silenced plants was much lower than that in mock plants (Fig 4B). *NbSKη* knock-down in *N*. *benthamiana* resulted in dwarf statue and extended petioles compared to mock-treated and wild-type plants, reminiscent of the long bending petiole induced by PVX-mediated expression of C4 (Fig 4C–4E). To further confirm the results from the transient expression system, we obtained the stable *NbSKη* knock-down transgenic *N*. *benthamiana* (*NbSKη-*RNAi) plants through RNAi technology. qRT-PCR analysis revealed that the transcripts of *NbSKη* were significantly reduced in *NbSKη-*RNAi plants compared with the amount in wild-type plants (Fig 4F). Again, the longer petioles were observed in *NbSKη-*RNAi plants as that in *35S*::*C4* transgenic plants (Fig 4G). Interestingly, both *NbSKη-*RNAi plants and *35S*::*C4* transgenic plants showed the downward leaf curling phenotype, one of the characteristic TLCYnV-induced symptoms (Fig 4F). Thus, all genetic results suggest that NbSKη likely plays important roles in C4-induced symptoms.

Previous work has shown that *N*. *benthamiana* constitutively expressing TLCYnV C4 developed virus-like downward leaf curling symptoms [11]. Similar phenotypes could also be shown in *A*. *thaliana* constitutively expressing TLCYnV C4. Notably, such leaf morphology phenocopied the transgenic *A*. *thaliana* plants overexpressing BZR1-CFP (Fig 4H). Importantly, the downward leaf curling phenotype of the transgenic *A*. *thaliana* plants expressing TLCYnV C4 also phenocopies that of the triple *GSK3* mutant (a triple knockout mutant for group II GSK3s) [44]. The genetic evidence suggests that C4 might alter the BR pathway. To test this hypothesis, we examined the phosphorylated/unphosphorylated level of BZR1 using *pBZR1*::*BZR1-CFP A*. *thaliana* with or without C4. Indeed, the level of unphosphorylated BZR1 was increased when C4 was transiently expressed (Fig 4I). We next used qRT-PCR analysis to detect the transcript levels of BR-targeted genes, *CPD* and *DWF4*, in wild-type, *35S*::*C4* transgenic, or *pBZR1*::*BZR1-CFP* transgenic *A*. *thaliana* plants. As expected, the expression of both *CPD* and *DWF4* genes was repressed in *35S*::*C4* and *pBZR1*::*BZR1-CFP* transgenic *A*. *thaliana* plants (Fig 4J). We confirmed that TLCYnV C4 could interact with AtSKη*/*BIN2 but not with BZR1 (S7 and S8 Figs). Together, the results suggest that TLCYnV C4 interrupts the BR signal transduction pathway to induce abnormal development through interacting with AtSKη*/*BIN2 in *A*. *thaliana*.

### TLCYnV C4 induces abnormal cell division through NbSKη-mediated BR signaling pathway in *N*. *benthamiana*

**Fig 5:**
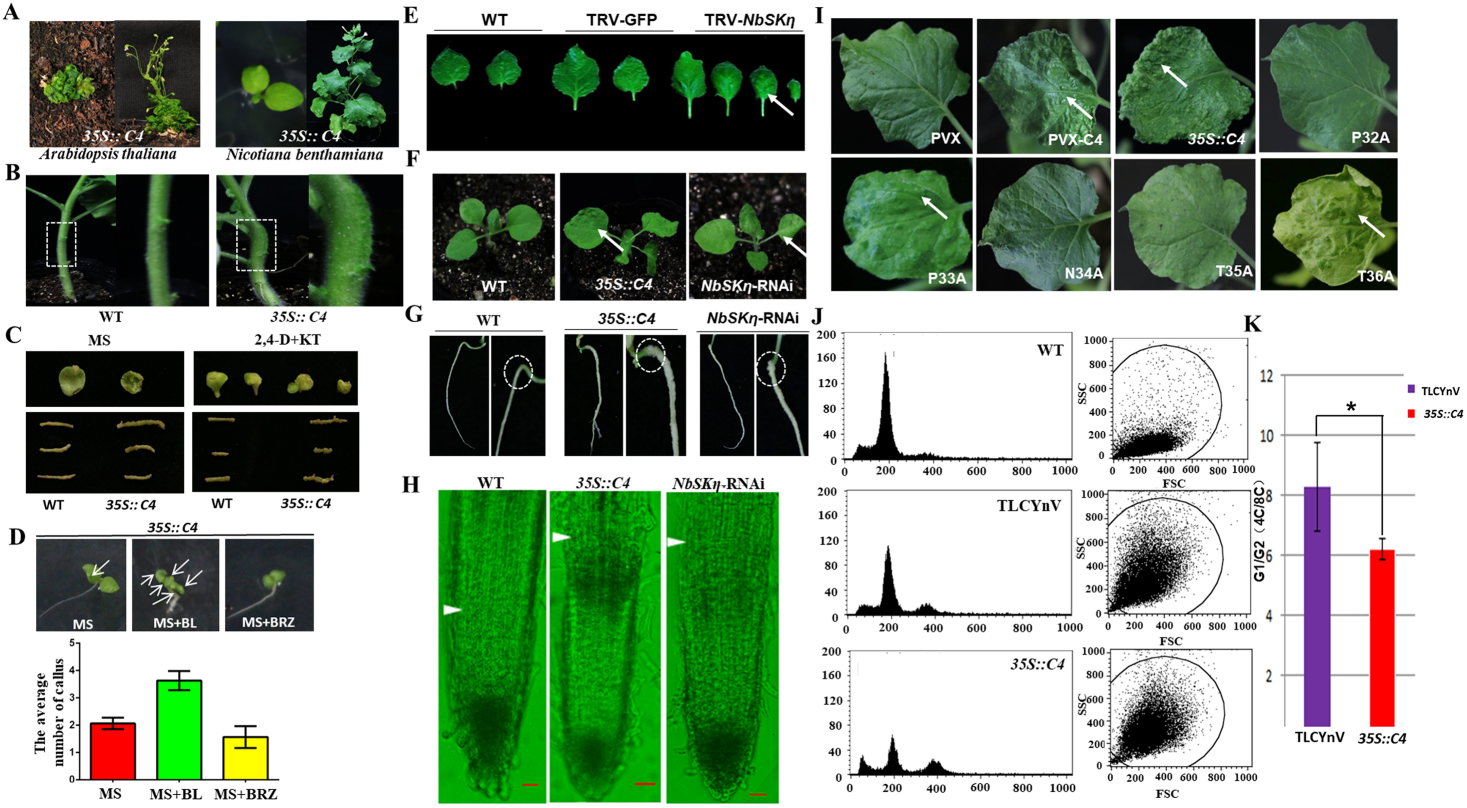
TLCYnV C4 induces the abnormal cell division through NbSKη-mediated BR pathway in *N*. *benthamiana*. **(A)** The phenotype of *35S*::*TLCYnV C4* transgenic *A*. *thaliana* (left panel) or *N*. *benthamiana* (right panel) plants. **(B)** Callus-like tissues could be found on the stem of *35S*::*TLCYnV C4* transgenic *N*. *benthamiana* plants. **(C)** Root and leaf callus formation in wild-type (WT) and *35S*::*TLCYnV C4* (*35S*::*C4*) transgenic *N*. *benthamiana* plants. The leaves of 14-day-old plants and the roots of 12-day-old plants were cut off and transferred on MS medium containing 100 ng/L 2,4-D and 300 ng/L kinetin (KT). Photographs were taken 14 days later. **(D)** The influence of BL or BRZ on the average number of callus-like tissue per leaf. **(E)** Leaf phenotype of WT, TRV-GFP inoculated, or TRV-*NbSKη* inoculated (*NbSKη-*silenced) plants. Arrows indicate the callus-like tissues. **(F)** Leaf phenotype of WT, *35S*::*C4*, or *NbSKη*-RNAi transgenic *N*. *benthamiana* plants. Arrows indicate the callus-like tissues. **(G)** Root phenotype of WT, *35S*::*C4*, or *NbSKη*-RNAi transgenic *N*. *benthamiana* plants. Circles indicate the callus-like tissues. The plants were grown on MS medium, photographs were taken at 14 days after sowing. **(H)** Meristem size of 14-day-old WT, *35S*::*C4* transgenic, or *NbSKη*-RNAi transgenic *N*. *benthamiana* plants. Arrowheads indicate the boundary between the proximal meristem and the elongation zone of the root. Scale bar = 50 μm. **(I)** The phenotype of *N*. *benthamiana* mature leaves inoculated with PVX, PVX-C4, or PVX-C4 mutants at 21 dpi. Arrows indicate the callus-like tissues. **(J)** Flow cytometric analysis of the cell ploidy under different treatments. **(K)** The cell percentage of different phase distribution in *35S*::*C4* transgenic *N*. *benthamiana* plants, or *N*. *benthamiana* plants inoculated with TLCYnV. * represents significant difference (P value <0.05).

To further study how C4 induced abnormal development, we pursued the stable transgenic plants expressing C4 (*35S*::*TLCYnV C4*). Interestingly, we observed callus-like tissues on the leaves and the stems of *35S*::*C4* transgenic plants (Fig 5A and 5B). To examine whether TLCYnV C4 accounted for the abnormal cell division, we conducted *in vitro* cell regeneration assays. We could only detect calli on the segments of *35S*:: *TLCYnV C4* transgenic *N*. *benthamiana* but not on those of wild-type plants at 12 days after *in vitro* culture. When the segments were cultured on MS medium supplemented with 2,4-dichlorophenoxyacetic acid (2,4-D) and kinetin (KT), calli could be induced on both wild-type and *35S*::*C4* transgenic segments. However, callus growth was enhanced on *35S*::*C4* transgenic segments compared to that on the wild-type segments (Fig 5C). These results demonstrate that cell division could be induced by TLCYnV C4.

BL, the most biologically active brassinosteroid, is a reliable chemical for study the regulation of cell division. BL could efficiently promote cell division in tobacco BY2 cells (S9 Fig). To examine whether C4 promotes the formation of callus-like tissues through the BR signal transduction pathway, wild-type and *35S*::*C4* transgenic *N*. *benthamiana* plants were cultured on MS medium with or without BL or the BR biosynthetic inhibitor brassinazole (BRZ). Notably, no significant difference on the rate of symptom appearance was detected between plants under different treatments (S10 Fig). However, the callus-like tissue formation was enhanced on *35S*::*C4* transgenic plants cultured on MS medium supplemented with BL compared to that on MS with or without BRZ (Fig 5D). This result strongly suggests that TLCYnV C4 indeed functions through interfering with the BR pathway.

If TLCYnV C4 impacts BR pathway via interacting with NbSKη, it is expected that NbSKη would also be involved in C4-induced callus-like cellular progress. Silencing *NbSKη* in *N*. *benthamiana* through TRV vector could promote formation of callus-like tissues (Fig 5E), supporting our hypothesis above. To further examine whether C4 regulated cell division genetically through NbSKη, we first compared the phenotype of *NbSKη-*RNAi, *35S*::*C4*, and wild-type plants. Both *35S*::*C4* and *NbSKη-*RNAi plants showed the callus-like tissues in their leaves and roots (Fig 5F and 5G). Moreover, the meristem size of both *35S*::*C4* and *NbSKη-*RNAi plants were obviously larger than that of wild-type plants. The boundary between the proximal meristem and the elongation zone of the root was delayed in the root tip of *35S*::*C4* and *NbSKη-*RNAi plants compared to that of wild-type plants (Fig 5H). The clear similarity of *35S*::*C4* and *NbSKη-*RNAi plants further supported that TLCYnV C4 targets NbSKη *in vivo*. To elucidate whether the direct interaction of C4/NbSKη is essential for the callus-like tissues formation, we used PVX-based expression system to express TLCYnV C4, and its mutants. *N*. *benthamiana* plants infiltrated with PVX-C4, PVX-C4(P33A) or PVX-C4(T36A) showed callus-like tissues in their leaves at 21 dpi. In contrast, the phenotype of leaves of *N*. *benthamiana* infiltrated with NbSKη-interaction compromised mutants, was similar to the one infiltrated with PVX alone at 21 dpi (Fig 5I). These results suggest the direct interaction between C4 and NbSKη is essential for the induction of cell division in *N*. *benthamiana*.

To further investigate how TLCYnV C4 altered cell cycle progress, we performed flow cytometric analysis. We isolated the nuclei from wild-type *N*. *benthamiana*, *35S*::*C4* transgenic *N*. *benthamiana*, or *N*. *benthamiana* infiltrated with TLCYnV infectious clone at 14 dpi to analyze the cell ploidy. We observed that the proportion of nuclei with 8C DNA content is obviously increased in *35S*::*C4* transgenic and TLCYnV-infected *N*. *benthamiana* plants compared to that in wild-type plants (Fig 5J). These results suggest that C4 expression or TLCYnV infection reactivates DNA replication. We also examined the proportion of nuclei with 8C DNA content in total nuclei isolated from the leaves under different treatments, consistent with flow cytometric analysis, the proportion of nuclei with 8C DNA content was found to be higher in *35S*::*C4* transgenic *N*. *benthamiana* plants than that in *N*. *benthamiana* plants infected with TLCYnV (Fig 5K). These results suggest that the existence of TLCYnV C4 might reduce the proportion of cells in G1 phase.

### NbSKη interacts with and phosphorylates NbCycD1;1

**Fig 6:**
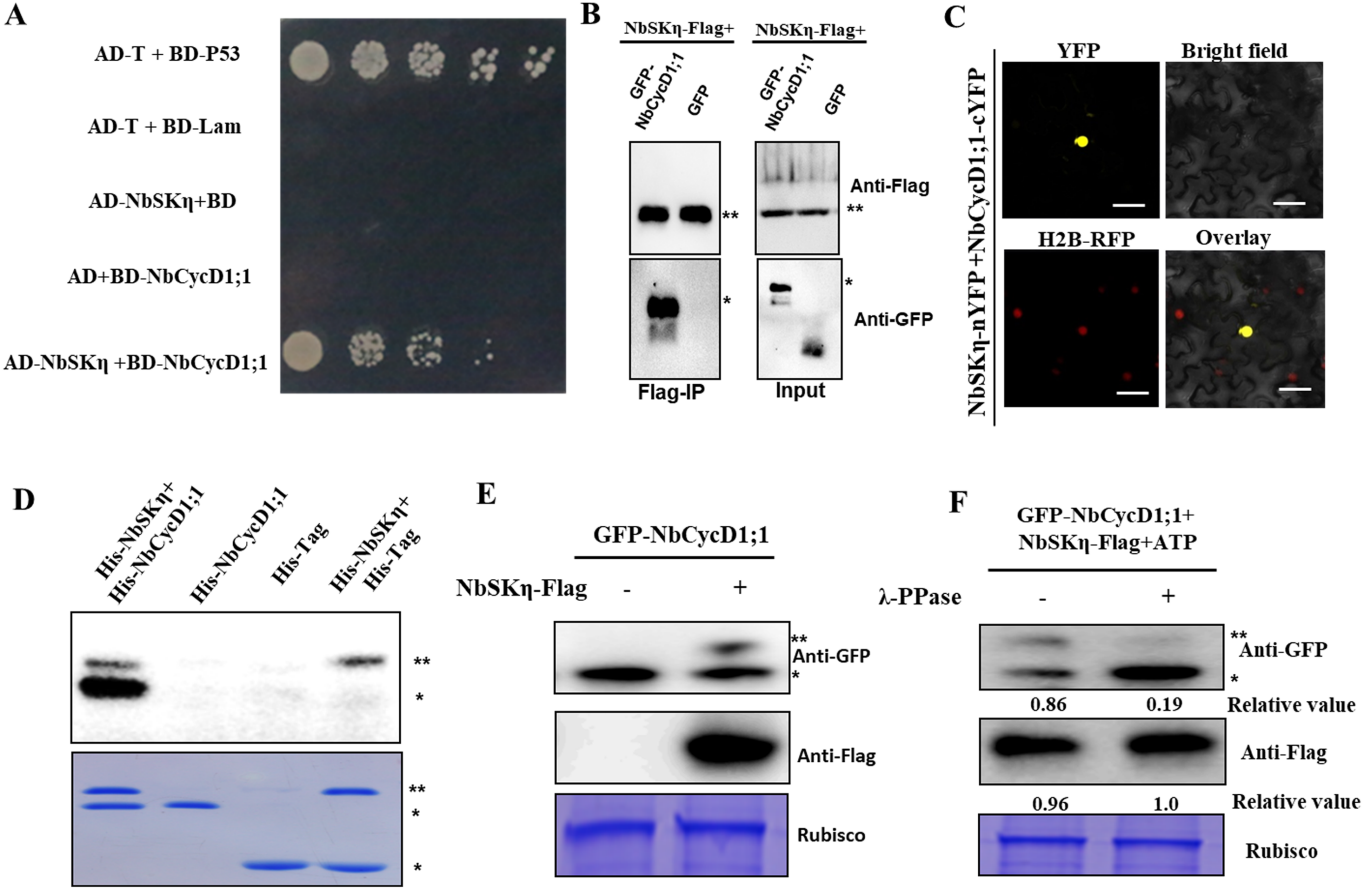
NbSKη interacts with and phosphorylates NbCycD1;1. **(A**-**C)** Interaction between NbCycD1;1 and NbSKη was validated in Y2H (A), Co-IP (B) and BiFC (C) assays. ** indicates NbSKη-Flag. * represents the substrate of NbSKη. **(D)** NbSKη phosphorylates NbCycD1;1 *in vitro*. The upper panel shows autoradiography and the bottom panel shows coomassie blue staining. ** indicates His-NbSKη. * represents the substrate of NbSKη. **(E)** NbSKη phosphorylates NbCycD1;1 *in vivo*. ** indicates phosphorylated form of GFP-CycD1;1. * represents the non-phosphorylated form of GFP-CycD1;1. **(F)** Identification of the phosphorylation of NbCycD1;1 mediated by NbSKη using λ-PPase. Plant tissues expressing GFP-NbCycD1;1 with or without Flag-NbSKη were extracted at 60 hpi. Two aliquots of samples were treated in the reaction system in the absence or presence of λ-PPase for only 10 min at 30°C. ** indicates phosphorylated form of GFP-CycD1;1. * represents the non-phosphorylated form of GFP-CycD1;1. Relative values represent the ratio of phosphorylated form and non-phosphorylated form of NbCycD1;1 (top panel) and the accumulation level of NbSKη-Flag (middle panel), respectively.

D-type cyclins regulate the G1/S-phase transition in plants and overexpression of D-type cyclins reduces the proportion of cells in G1 phase [41, 45–47]. Also, GSK3β is reported to phosphorylate and regulate cyclin D1 proteolysis in animal [48]. So, we tested whether NbSKη regulates cell division by targeting NbCycD1;1. NbCycD1;1 is a homologue of NtCycD1;1 and shares 95% identity with NtCycD1;1 (XP 016457049.1). Using Y2H and co-immunoprecipitation assays, we found that NbSKη could interact with NbCycD1;1 (Fig 6A and 6B). Furthermore, we infiltrated H2B-RFP transgenic *N*. *benthamiana* with a construct expressing GFP-NbCycD1;1, the GFP fluorescence was observed at 60 hpi, we found that GFP signal of GFP-NbCycD1;1 could only be detected in the nucleus (S11 Fig). BiFC assay showed that the interaction between NbSKη and NbCycD1;1 occurred only in the nucleus (Fig 6C).

Interaction of NbCycD1;1 with NbSKη raised the possibility that NbCycD1;1 is the substrate of NbSKη. To test this hypothesis, we conducted *in vitro* phosphorylation assays. We purified His-NbSKη, and His-NbCycD1;1 from *E*. *coil*. His-NbCycD1;1 was indeed phosphorylated by His-NbSKη *in vitro* (Fig 6D). To examine whether NbSKη could phosphorylate NbCycD1;1 *in vivo*, we transiently expressed GFP-NbCycD1;1 with or without Flag-NbSKη in *N*. *benthamiana*. Tricine SDS-PAGE and western blot results showed that a phosphorylated form of GFP-NbCycD1;1 could be detected in the presence of Flag-NbSKη (Fig 6E). To further validate the top-shifted band was indeed phosphorylated form of GFP-NbCycD1;1, we treated the protein extracts with λ-protein phosphatase (λ-PPase). Interestingly, λ-PPase could decrease the level of phosphorylated GFP-NbCycD1;1 (Fig 6F). These results suggested that NbSKη could phosphorylate NbCycD1;1 *in vitro* and *in vivo*.

### Phosphorylation of NbCycD1;1 mediated by NbSKη promotes proteasomal degradation of NbCycD1;1

**Fig 7:**
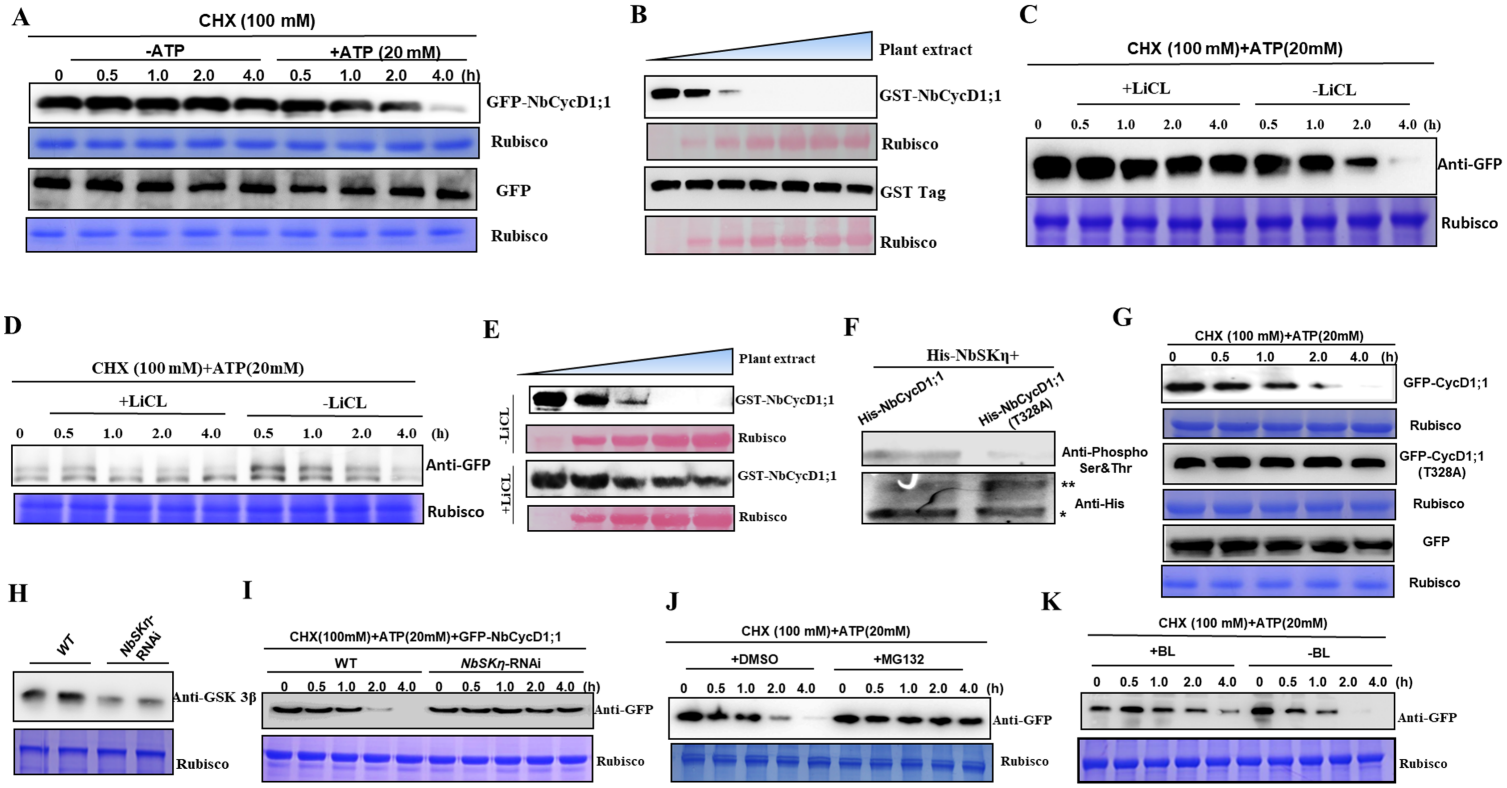
Phosphorylation of NbCycD1;1 mediated by NbSKη promotes NbCycD1;1 degradation via 26S proteasome. **(A)** Detection of the stability of GFP or GFP-NbCycD1;1 protein by semi-*in vivo* assays. GFP or GFP-NbCycD1;1 protein was analyzed with anti-GFP antibody at different time points after CHX treatment in the presence or absence of ATP. **(B)** Detection of the stability of GST or GST-NbCycD1;1 protein by *in vitro* assays. Purified GST or GST-NbCycD1;1 was mixed with additive volumes of fresh plant extract and then diminishing volumes of elution buffer were added for the identical concentration of fusion proteins at 25°C for 2h. GST or GST-NbCycD1;1 protein level was analyzed with anti-GST antibody. **(C** and **D)** Tris SDS-PAGE (C) or Tricine SDS-PAGE (D) analysis of the influence of NbCycD1;1 phosphorylation mediated by NbSKη on NbCycD1;1 stability by semi-*in vivo* assays. **(E)** Detection of the influence of NbCycD1;1 phosphorylation mediated by NbSKη on NbCycD1;1 stability by *in vitro* assays. **(F)** The phosphorylation level of NbCycD1;1 or NbCycD1;1 (T328A) by NbSKη *in vitro*. ** indicates His-NbSKη. * represents the substrate of NbSKη. **(G)** Western blot analysis of stability of GFP-NbCycD1;1, GFP-NbCycD1;1 (T328A), or GFP by semi-*in vivo* assays. **(H)** Western blot analysis of the protein level of NbSKη in wild-type (WT) or *NbSKη-*RNAi plants. **(I)** Western blot analysis of the stability of GFP-NbCycD1;1 in WT or *NbSKη-*RNAi *N*. *benthamiana* plants by semi-*in vivo* assays. **(J)** Effect of MG132 on the stability of GFP-NbCycD1;1 protein by semi-*in vivo* assays. GFP-NbCycD1;1 protein levels were analyzed with anti-GFP antibody at different time points after 100 μM CHX and 20 mM ATP treatments in the presence of 100 μM MG132 or an equal volume of DMSO (control). **(K)** Effect of BL on the stability of GFP-NbCycD1;1 protein in semi-*in vivo* assays.

Because D-type cyclins exert time-specific functions in cell cycle, we detected the stability of NbCycD1;1. We infiltrated *N*. *benthamiana* with GFP-NbCycD1;1 or GFP. Total protein extracts were prepared at 60 hpi, and GFP-NbCycD1;1 or GFP level was analyzed with anti-GFP antibody at different times after treating with cycloheximide (CHX) in the presence or absence of ATP. The results showed that GFP-NbCycD1;1, different from GFP alone, could be readily degraded in cell extracts in the presence of ATP, but not in extracts lacking ATP (Fig 7A).

To further examine the stability of NbCycD1;1, we expressed the GST-NbCycD1;1 or GST prokaryotically, and purified GST-NbCycD1;1 or GST. GST-NbCycD1;1 or GST protein level was analyzed with anti-GST antibody after mixing with different volumes of fresh plant extract for 2h at 25°C. The results showed that GST-NbCycD1;1 was more stable in the mixture harboring less plant extract. However, GST protein was still stable in mixture harboring different volumes of fresh plant extract (Fig 7B).

We next examined the influence of NbSKη-catalyzed NbCycD1;1 phosphorylation on the stability of NbCycD1;1. We expressed GFP-NbCycD1;1 in *N*. *benthamiana* plants, and total protein extracts were treated with or without GSK3 inhibitor LiCl in the presence of ATP and CHX (Fig 7C). Tricine SDS-PAGE and western blot results showed the non-phosphorylated NbCycD1;1 was stable, and phosphorylated NbCycD1;1 could be readily degraded in the presence of LiCl (Fig 7D). These results were easily reproducible in the semi-*in vitro* assays with recombinant proteins (GST-NbCycD1;1) from *E*. *coil* and cell extracts from *N*. *benthamiana* plants (Fig 7E). These results suggest that NbCycD1;1-mediated NbCycD1;1 phosphorylation promotes its degradation.

To identify the phosphorylation site of NbCycD1;1 by NbSKη, we used phosphorylation system *in vitro* to look for the key point(s) of NbCycD1;1 important for NbSKη phosphorylation. Phosphorylated NbCycD1;1 was analyzed with anti-phosphoserine/threonine antibody after reacting with NbSKη in the kinase reaction system for 30 min. The result showed that NbCycD1;1 could be phosphorylated by NbSKη and NbCycD1;1(T328A) mutant could not be phosphorylated by NbSKη (Fig 7F). This result indicated that T328 of NbCycD1;1 was a key phosphorylation site of NbCycD1;1 by NbSKη. In consistent with the *in vitro* assay, GFP-NbCycD1;1(T328A) was more stable than GFP-NbCycD1;1 in the presence of ATP (Fig 7G).

To further examine whether phosphorylation of NbCycD1;1 mediated by NbSKη promotes the degradation of NbCycD1;1, we pursued *NbSKη-*RNAi transgenic *N*. *benthamiana* plants. The protein level of NbSKη in wild-type or *NbSKη-*RNAi transgenic plants was analyzed with anti-GSK 3β antibody by western blot and the result showed that NbSKη protein level was much higher in wild-type plants than that in *NbSKη-*RNAi plants (Fig 7H). We expressed GFP-NbCycD1;1 transiently in wild-type plants or *NbSKη-*RNAi transgenic plants. Western blot results showed that GFP-NbCycD1;1 was much more stable in *NbSKη-*RNAi transgenic plants than that in wild-type plants (Fig 7I). Notably, treatment of MG132 could stabilize the GFP-NbCycD1;1 protein (Fig 7J). This result indicates that NbCycD1;1 is degraded via 26S proteasome.

Because BL could inactivate SHAGGY-like kinase η (SKη) in BR signal transduction pathway, we tested the stability of NbCycD1;1 with or without BL treatment in *semi-in vivo* degradation assays. Western blot showed that BL treatment enhanced the stability of NbCycD1;1 (Fig 7K). This result indicates that phosphorylation of NbCycD1;1 by NbSKη promotes its degradation.

### TLCYnV C4 inhibits its proteasomal degradation of NbCycD1;1 by interacting with NbSKη

**Fig 8:**
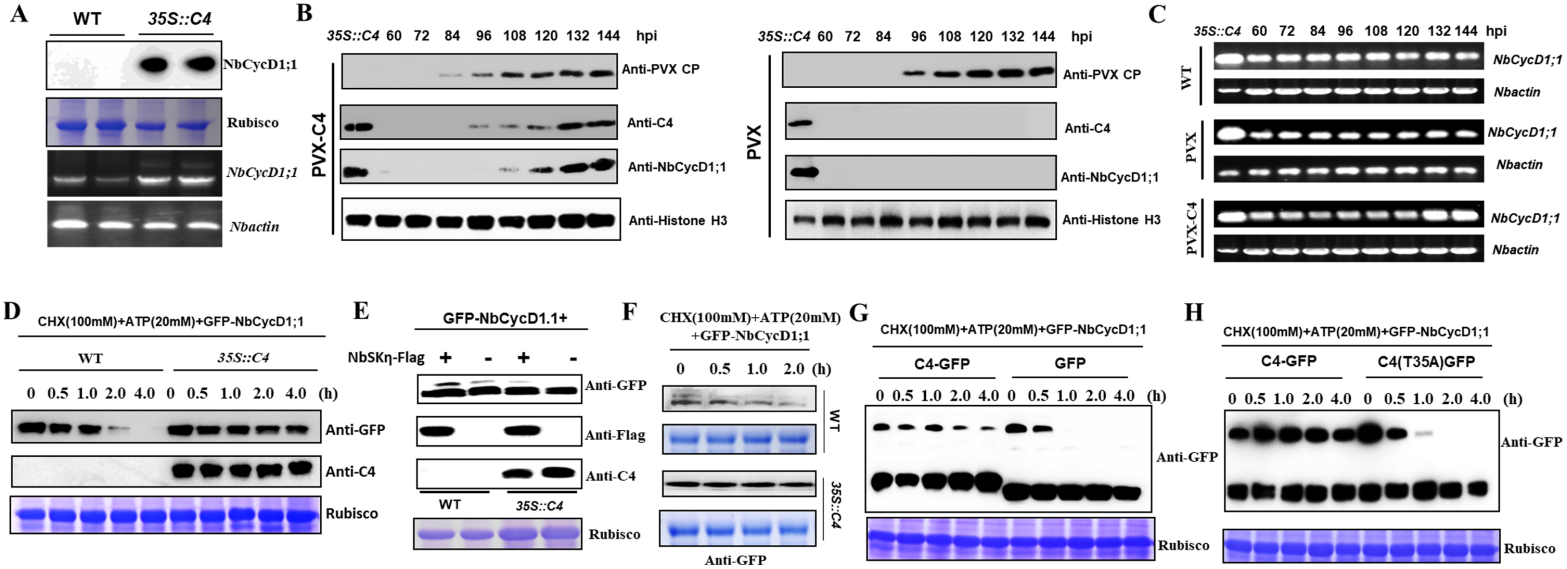
TLCYnV C4 inhibits proteasomal degradation of NbCycD1;1 by interacting with NbSKη. **(A)** The protein and transcript levels of NbCycD1;1 in wild-type (WT) and *35S*::*C4* transgenic *N*. *benthamiana* plants. Upper panel shows the protein level of NbCycD1;1 and lower panel shows transcript level of *NbCycD1;1* in wild-type and *35S*::*C4* transgenic *N*. *benthamiana* plants. **(B)** Time-course analysis of the NbCycD1;1 protein level in *N*. *benthamiana* plants inoculated with PVX or PVX-C4 at different time points. **(C)** Time-course analysis of the NbCycD1;1 transcript level in *N*. *benthamiana* plants inoculated with PVX or PVX-C4 at different time points. **(D)** Western blot analysis of the stability of GFP-NbCycD1;1 in WT and *35S*::*C4* transgenic *N*. *benthamiana* plants by semi-*in vivo* assays. **(E)** Tricine SDS-PAGE analysis of the accumulation of phosphorylated/nonphosphorylated GFP-NbCycD1;1 in WT and *35S*::*C4* transgenic *N*. *benthamiana* plants with or without NbSKη. **(F)** Tricine SDS-PAGE analysis of the stability of GFP-NbCycD1;1 in WT and *35S*::*C4* transgenic *N*. *benthamiana* plants. **(G)** Western blot analysis of the satbility of GFP-NbCycD1;1 in *N*. *benthamiana* tissues co-expressing GFP-NbCycD1;1 with GFP or C4-GFP by semi-*in vivo* assays. **(H)** Western blot analysis of the stability of GFP-NbCycD1;1 in *N*. *benthamiana* tissues co-expressing GFP-NbCycD1;1 with C4-GFP or C4(T35A)-GFP by semi-*in vivo* assays.

The restriction of NbSKη by TLCYnV C4 raised the possibility that C4 might stabilize the NbCycD1;1. To test this hypothesis, we analyzed accumulation level of NbCycD1;1 in wild-type or *35S*::*C4* transgenic *N*. *benthamiana* plants with anti-NbCycD1;1 antibody. We found that the accumulation level of NbCycD1;1 protein was higher in *35S*::*C4* transgenic plants than that in wild-type plants. However, the transcript level of *NbCycD1;1* in *35S*::*C4* transgenic plants was also higher than that in wild-type plants (Fig 8A). To further identify whether TLCYnV C4 could stabilize NbCycD1;1 *in vivo*, we inoculated *N*. *benthamiana* with the PVX-based vector or the PVX-based vector harboring *C4* gene. Total protein or RNA samples were extracted at different time points. Western blot results showed that NbCycD1;1 protein could be detected at 108 hpi in plants inoculated with PVX-C4, but not in plants inoculated with PVX. We also used semi-quantitative RT-PCR to analyze the transcript accumulation levels of *NbCycD1;1* at different time points. Interestingly, significant increase of *NbCycD1;1* transcript level appeared at 132 hpi in plants inoculated with PVX-C4. Whereas steady-state levels of *NbCycD1;1* remained stable at different time points in wild-type plants or plants inoculated with PVX. Notably, there is no significant differences on *NbCycD1;1* transcript level between 96 hpi and 120 hpi in plants inoculated with PVX-C4, however, increased NbCycD1;1 protein accumulation level could be detected in this period (Fig 8B and 8C). These results suggest that TLCYnV C4 inhibits the degradation of NbCycD1;1 *in vivo*.

To further examine how TLCYnV C4 affected the half-life of NbCycD1;1, we inoculated wild-type*a* or *35S*::*C4* transgenic *N*. *benthamiana* plants with agrobacteria expressing GFP-NbCycD1;1. Total protein samples were extracted at 60 hpi, and GFP-NbCycD1;1 was analyzed with anti-GFP antibody after treating with CHX and ATP. Western blot results showed that GFP-NbCycD1;1 protein was much more stable in *35S*::*C4* transgenic *N*. *benthamiana* than that in *N*. *benthamiana* (Fig 8D). To further study whether C4 altered the level of phosphorylated NbCycD1;1, we expressed GFP-NbCycD1;1 with or without Flag-NbSKη in wild-type or *35S*::*C4* transgenic *N*. *benthamiana* plants. Tricine SDS-PAGE analysis showed that the accumulation level of phosphorylated NbCycD1;1 was much higher in *N*. *benthamiana* than that in *35S*::*C4* transgenic plants. When GFP-NbCycD1;1 was co-expressed with Flag-NbSKη in wild-type or *35S*::*C4* transgenic *N*. *benthamiana* plants, the accumulation level of phosphorylated NbCycD1;1 was much higher in wild-type than that in *35S*::*C4* transgenic *N*. *benthamiana* plants (Fig 8E). We used semi-in vivo degradation assays to detect the accumulation level of GFP-NbCycD1;1 in wild-type or *35S*::*C4* transgenic *N*. *benthamiana* plants. Tricine SDS-PAGE analysis showed that phosphorylated NbCycD1;1 could be detected in wild-type *N*. *benthamiana* plants and the phosphorylated NbCycD1;1 was degraded within one hour. No phosphorylated NbCycD1;1 could be detected in *35S*::*C4* transgenic plants and NbCycD1;1 was stable in *35S*::*C4* transgenic plants (Fig 8F). These results suggest that TLCYnV C4 inhibits NbCycD1;1 phosphorylation to stabilize NbCycD1;1. We also repeated this assay with a C4 mutant, and observed that GFP-NbCycD1;1 was much more easily degraded in plants expressing GFP-NbCycD1;1/C4(T35A)-GFP or GFP-NbCycD1;1/GFP than that in plants expressing GFP-NbCycD1;1/C4-GFP (Fig 8G and 8H). To further identify whether NbCycD1;1 plays an important role for C4-induced cell division, we silenced *NbCycD1;1* gene using the TRV-based vector, then inoculated PVX or PVX-C4 in knock-down and mock plants. The results showed that less callus-like tissues were observed in *NbCycD1;1* knock-down plants than in mock plants at 21 dpi (S12A and S12B Fig). All together, these results indicate that TLCYnV C4 stabilizes NbCycD1;1 via the sequestering of NbSKη to the membrane to prevent NbCycD1;1 from phosphorylation.

## Discussion

Previous studies have demonstrated that several geminiviruses AC4 or C4 could interfere the plant development through targeting AtSKη and AtSKζ [12]. In this study, we re-discovered that TLCYnV C4 targets NbSKη, and further pinpointed a few critical residues exemplified by P32, N34 and T35 in C4 that are engaged in the interaction. We reported that TLCYnV C4 determination of the symptom entails its direct interaction with NbSKη.

How does the interaction of TLCYnV C4 with NbSKη lead to symptom? Our results indicate that NbSKη is localized mainly in the nucleus. However, the presence of TLCYnV C4 alters the localization pattern of NbSKη, recruits NbSKη to the membrane and decreases the accumulation level of NbSKη in the nucleus. The scenario that C4 sequsters NbSKη to a particular cellular niche is reminiscent of earlier observations with different viruses. TMV infection revealed that the chloroplast-localized NRIP1 (N receptor-interacting protein) could be translocated to cytoplasm and nucleus by the helicase domain of TMV 183 kDa protein [49]. The disease-specific protein (SP) of RSV could alter the distribution pattern of PsbP in tobacco and rice [50]. Trapping of NbSKη into membrane interferes the NbSKη-mediated BR pathway in plants. This model is supported by several pieces of genetic evidence: (1) the phenotype of transgenic plants (*A*. *thaliana*) expressing TLCYnV C4 under the control of CaMV 35S promoter mimicked that of BZR1 over-expression plants. (2) *NbSKη-*silenced *N*. *benthamiana* plants showed the longer petiole phenotype as observed in the *N*. *benthamiana* plants inoculated with PVX-C4. (3) In the presence of TLCYnV C4, the level of phosphorylated BZR1 was decreased in *A*. *thaliana*. (4) The expression of BZR1-targeted genes, *CPD* and *DWF4*, were repressed in both *35S*::*C4* and *pBZR1*::*BZR1-CFP* transgenic *A*. *thaliana* plants. However, TLCYnV C4 does not interact with BZR1 in Y2H assays (S8 Fig). We believed that TLCYnV C4 influences BR signal transduction pathway through interacting with BIN2.

We found that NbSKη could interact with and phosphorylate NbCycD1;1, the phosphorylation of NbCycD1;1 promotes its proteasomal degradation in *N*. *benthamiana* plants. This is likely the reason that the protein accumulation level of NbCycD1;1 is extremely low in mature leaves. NbCycD1;1 localizes only in the nucleus. However, C4 could decrease the accumulation level of nucleus-localized NbSKη, so NbSKη could not interact with and phosphorylate NbCycD1;1 in the nucleus, the protein level of NbCycD1;1 is increased, leading to the induction of cell division and formation of the callus-like tissues. The physiological advantage for such induction of cell division could make an environment suitable for DNA virus replication. In line with this model, we observed that virus accumulation level of TLCYnV was higher in *NbSKη-*RNAi transgenic plants than that in wild-type *N*. *benthamiana* plants (S13 Fig). In this context, we favor an idea that TLCYnV C4 could stabilize NbCycD1;1 protein through relocating NbSKη. However, we have no reason to exclude other possibilities: (1) Because both NbCycD1;1 and TLCYnV C4 could interact with NbSKη and act as its substrates, C4 may compete for the phosphoryl group with NbCycD1;1, resulting stabilization of the latter. (2) *35S*::*C4* transgenic *N*. *benthamiana* plants phenocopy *35S*::*NbCycD1;1* transgenic *N*. *benthamiana* plants on callus-like tissue formation (S14A Fig). However, the number of callus-like tissues produced in *35S*::*C4* transgenic *N*. *benthamiana* plants mature leaves is more than those produced in mature leaves of *35S*::*NbCycD1;1* transgenic *N*. *benthamiana*. The accumulation level of NbCycD1;1 protein in *35S*::*NbCycD1;1* transgenic *N*. *benthamiana* is higher than that in *35S*::*C4* transgenic *N*. *benthamiana* plants (S14B Fig). NbSKη might interact with other cyclins that play different roles in cell cycle in the nucleus. Indeed NbSKη could also interact with NbCycD3;2, a homologue of NtCycD3;2 (NP 001312466.1), in the nucleus (S15A and S15B Fig), and NbCycD3;2 was more stable in *35S*::*C4* transgenic *N*. *benthamiana* plants than in wild-type *N*. *benthamiana* plants (S15C Fig). TLCYnV C4 might stabilize them to promote cell cycle. Further effort to the above hypothesis might provide new insights into how C4 alters cell cycle.

In plant, we found that NbSKη could phosphorylate NbCycD1;1 to promote the degradation of NbCycD1;1. Our work is consistent with the previous studies that phosphorylation of D-type cyclin by GSK 3β triggers its proteasome-dependent degradation in animal [48, 51, 52], however, Yang et al. reported that GSK3β has a limited role in cell cycle regulation of cyclin D1 levels in animal [53]. More functional studies on GSK 3β are necessary for both plant and animal to clarify this difference.

Previous studies demonstrated that beet curly top virus (BCTV) C4 binds to the plasma membrane [16]. Here is the same case with TLCYnV C4. C4 mutants (P32A, N34A, or T35A) that could not interact with NbSKη could enter the nucleus stably (S6A Fig). This suggests that the interaction between TLCYnV C4 and NbSKη may promote the nuclear export of C4/NbSKη complex. The reason that the presence of TLCYnV C4 in the nucleus could lead to the nuclear export of C4/NbSKη complex is likely due to the functional modification occurred on the specific site(s) of C4. For geminiviruses, several studies have shown that some viral proteins could be functionally phosphorylated by the specific kinases. SlSnRK1 phosphorylation of AL2 delays cabbage leaf curl virus (CaLCuV) infection in *Arabidopsis* [54]. βC1 of TYLCCNV could also be phosphorylated by SlSnRK1 at Ser33 and Thr78 [55]. In particular, BCTV C4 and tomato golden mosaic virus C4 could be phosphorylated by AtSKη [12], and Ser49 of BCTV C4 could be phosphorylated in planta [16]. It is well known that a stable kinase-substrate docking interaction is essential for protein phosphorylation mediated by GSK3 kinases. BIN2 utilizes a direct kinase substrate docking mechanism for phosphorylation of BZR1, and BZR1 carries a portable docking motif to facilitate its phosphorylation by BIN2 [56]. Further effort for identification of potential phosphorylated site(s) on TLCYnV C4 that facilitates its phosphorylation by NbSKη might provide new insight on the molecular mechanism of how C4/NbSKη interaction promotes the nuclear export of C4/NbSKη complex.

It appears that the mechanism of C4-induced symptoms is different from that of TYLCCNV βC1 protein as TYLCCNV βC1 could not interact with AtSKη/BIN2 *in vivo* (S16 Fig). βC1, the pathogenicity factor of TYLCCNV, interacts with AS1 to alter leaf development [57]. Furthermore, the C4-induced symptoms were also different from those induced by TYLCCNV βC1 (S17 Fig). Other viral proteins, such as CaLCuV AL2 protein and tomato yellow leaf curl virus (TYLCV) V2 protein [58,59], which could not induce TLCYnV C4-like symptoms, were also found to not interact with AtSKη/BIN2 *in vivo* (S16 Fig).

**Fig 9:**
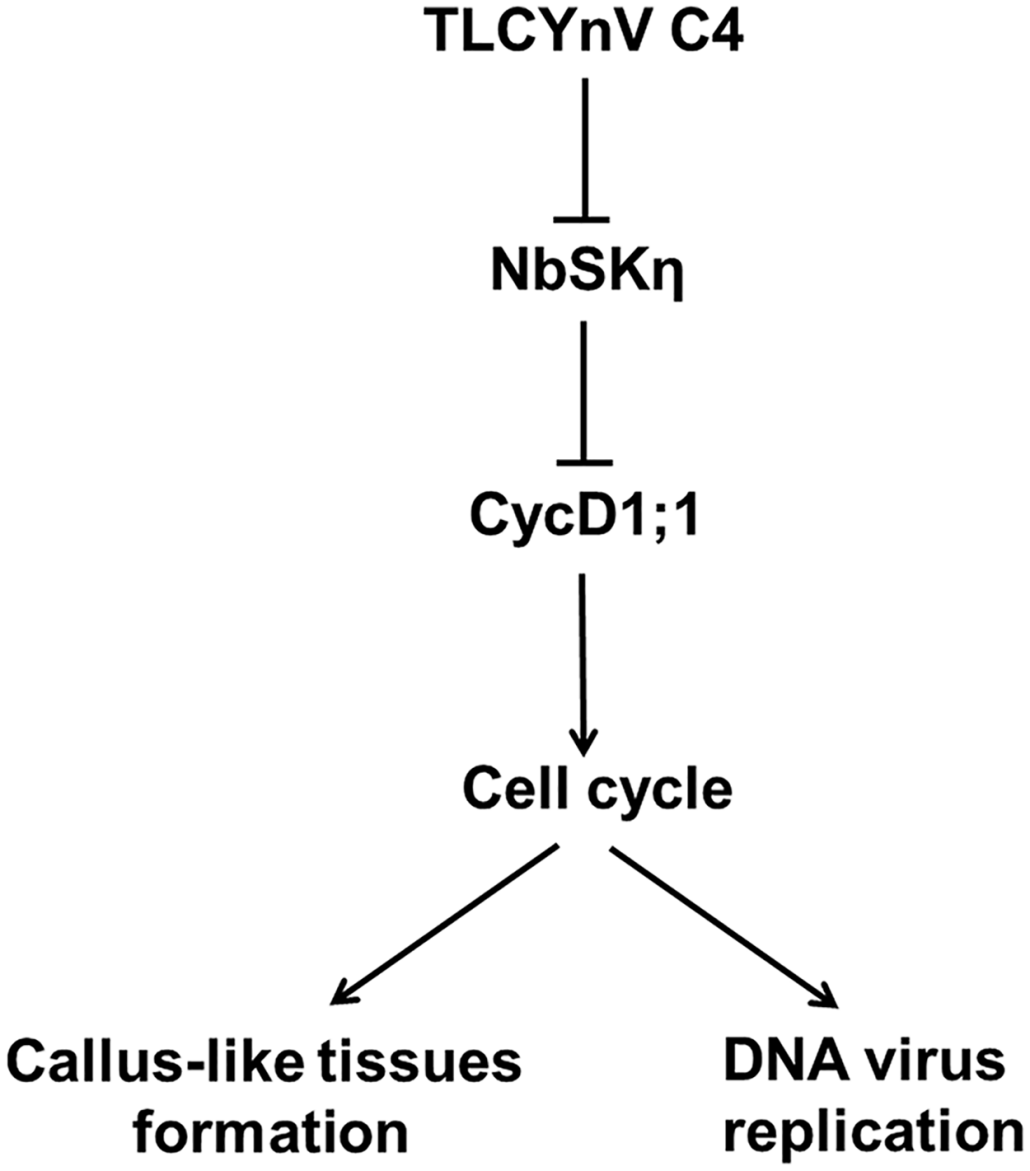
The proposed model to show how TLCYnV C4 interferes with the cell cycle in mature leaves through changing the nuclear-localization of NbSKη. TLCYnV C4 directs NbSKη to the cyto-membrane and reduces the accumulation of nucleus-localized NbSKη. The redistribution of NbSKη mediated by C4 interrupts the phosphorylation and proteasomal degradation of nucleus-located NbCycD1;1, and then increases the accumulation level of NbCycD1;1. Increased accumulation of NbCycD1;1 induces the callus-like tissues formation and creates an environment suitable for DNA virus replication in mature and differentiated leaves.

In summary, we provided the evidence that P32, N34 and T35 of TLCYnV C4 are important for its interaction with NbSKη, and the interaction has an effect on the C4-induced symptoms. C4 hijacks the NbSKη on the membrane to avoid the phosphorylation and proteasomal degradation of nucleus-located NbCycD1;1, and then increase the accumulation level of NbCycD1;1. The biological significance for this relocalization is to reactivate an environment suitable for DNA virus replication in mature and differentiated leaves and induce cell divisions (Fig 9).

## Materials and methods

### Plant material and growth conditions

Transgenic *N*. *benthamiana* plants expressing a nuclear marker-H2B-RFP (full-length red fluorescent protein fused to the C-terminus of histone 2B) [60] were kindly provided by Dr. Michael M. Goodin (University of Kentucky. KY, USA). Transgenic *A*. *thaliana* plants over-expressing BZR1-CFP (full-length cyan fluorescent protein fused to the C-terminus of BZR1) under the control of *BZR1* native promoter [29] were kindly provided by Dr. Joanne Chory (The Salk Institute for Biological Studies. CA, USA). Transgenic *N*. *benthamiana* plants expressing TLCYnV C4 were described [11]. *N*. *benthamiana* plants were grown inside a growth chamber at 26°C, under a 16-h light/8-h dark photoperiod. The *A*. *thaliana* ecotype Columbia (Col-0) was used as the wild-type plants for generating transgenic plants and phenotype comparison. The methods for seed sterilization and germination for plant growth were described in Li *et al* [61].

### Generation of transgenic plants

TLCYnV *C4* gene was PCR-amplified using primer pairs pCHF3-TLCYnV C4-F/R. The PCR fragment was cloned into binary vector (pCHF3) under the control of CaMV 35S promoter. The plasmid was transformed into *Agrobacterium tumefacience* strain EHA105. *A*. *thaliana* ecotype Col-0 plants transformed with the vector by the floral-dip method [62, 63]. The transgenic seeds were selected on standard MS medium [64] containing kanamycin (100 mg/L) and carbenicillin (250 mg/L). The antibiotic-resistant transformed plants were verified by PCR using primers used to make the expression construct.

### Plasmids construction

*NbSKη* was cloned into p2YN, pGADT7, pGD-Flag, pET-32a, and pGD-GFP vectors, and TLCYnV *C4* was cloned into p2YC, pGBKT7, pCHF3, pCHF3-GFP, pCHF3-CFP, pGD-Myc, pGD-Flag-Myc and pgR106 vectors. *NbCycD1;1* was cloned into pGBKT7, p2YC, pGD-GFP, pGEX4T-3, and pET-32a vectors. *NbCycD1;1(T328A)* was cloned into pGD-GFP and pET-32a vectors. *BIN2* and *BZR1* were cloned into pGADT7 vector individually, *BIN2* was cloned into p2YN and pGD-HA vectors. TYLCCNV *βC1*, CaLCuV *AL2* and TYLCV *V2* were cloned into pGD-Flag-Myc vector individually. The C4 N-terminal mutant I (27–85 aa), C4 N-terminal mutant II (44–85 aa), C4 Central-part mutant I (1–26 aa+62–85 aa), and C4 Central-part mutant II (1–26 aa+52–85 aa) were cloned individually into pGBKT7 vector. C4(P32A), C4(P33A), C4(N34A), C4(T35A), and C4(T36A) were cloned individually into pGBKT7 and pgR106 vectors. C4(P32A), C4(P33A), C4(N34A), C4(T36A), and C4(T36A) were cloned individually into pCHF3-GFP vector, C4(P32A), C4(P33A), C4(N34A), C4(T36A), and C4(T36A) were cloned into pCHF3-CFP vector. The part of the coding sequence of *NbSKη* was cloned into pTRV-RNA2 for virus-induced gene silencing (VIGS). All cDNAs were PCR-amplified using the KOD-Plus-Neo High-Fidelity DNA polymerase (TOYOBO). The resulting PCR fragments were first cloned individually into the pMD18-T vector (TaKaRa), then released from the vector using corresponding restriction enzymes. The released fragments were ligased into the expression vectors. C4 Central-part mutant I (1–26 aa+62–85 aa), C4 Central-part mutant II (1–26 aa+52–85 aa), C4(P32A), C4(P33A), C4(N34A), C4(T35A), and C4(T36A) were generated by the overlapping PCR methods as described previously [65]. All primers used for plasmids construction are listed in Supplemental Table 1 (S1 Table).

### Agroinfection assays in *N*. *benthamiana*

The constructs whose backbone is pgR106 were transformed into *A*. *tumefaciens* (strain GV3101) by electroporation, others were introduced into *A*. *tumefaciens* (strain C58C1) by electroporation. The transformed bacteria cultures were grown individually until approximately OD_600_ = 0.5~0.8. The cultures were collected and re-suspended using an induction buffer (10mM MgCl_2_, 100mM MES (pH 5.7), 2mM acetosyringone) for 3h at room temperature. The suspensions were adjusted to OD_600_ = 0.5. For co-localization experiments, the individual cultures were adjusted to OD_600_ = 1.0 and equal volumes were mixed before leaf infiltration. The suspensions were infiltrated into leaves of 4- to 6-week old *N*. *benthamiana* leaves using 1-ml needleless syringes.

### Y2H assay

The coding sequence of TLCYnV C4 was cloned into vector pGBKT7 that contains the GAL4 binding domain as instructed (Clontech). Construction and screen of a *N*. *benthamiana* cDNA library were performed as described in the BD Matchmaker Library Construction and Screening Kits User Manual (Clontech), and positive clones were selected on a synthetic medium lacking histidine and adenine, then confirmed by beta-X-gal assays.

The full-length *NbSKη* gene was PCR-amplified from *N*. *benthamiana* leaf cDNA, and then cloned into the pGADT7 vector (Clontech), which contains the GAL4 activation domain. The coding sequence of C4, C4 N-terminal mutant I (27–85 aa), C4 N-terminal mutant II (44–85 aa), C4 Central-part mutant I (1–26 aa+62–85 aa), C4 central-part mutant II (1–26 aa+52–85 aa), C4(P32A), C4(P33A), C4(N34A), C4(T35A), and C4(T36A) were cloned individually into the vector pGBKT7. Analyses the interactions were performed as described previously [50]. Combination of plasmids AD-NbSKη and BD-C4, AD- NbSKη and BD-C4 N-terminal mutant I (27–85 aa), AD-NbSKη and BD-C4 N-terminal mutant II (44–85 aa), AD-NbSKη and BD-C4 central-part mutant I (1–26 aa+62–85 aa), AD-NbSKη and BD-C4 central-part mutant II (1–26 aa+52–85 aa), AD-NbSKη and BD-C4(P32A), AD-NbSKη and BD-C4(P33A), AD-NbSKη and BD-C4(N34A), AD-NbSKη and BD -C4(T35A), or AD-NbSKη and BD-C4(T36A), were co-transformed into *Saccharomyces cerevisiae* strain Gold (Clontech). AD-T and BD-P53 were transformed into *S*. *cerevisiae* strain Gold to serve a positive control, AD-T and BD-Lam, AD and BD-C4, or AD-NbSKη and BD were co-transformed into *S*. *cerevisiae* strain Gold to serve a negative control. All transformants were grown at 30°C for 72h on the SD medium lacking Leu and Trp, and then transferred to the medium lacking Leu, Trp, His, and Ade.

### BiFC assay

The BiFC assay was performed on 5-week-old transgenic H2B-RFP *N*. *benthamiana* leaves infiltrated with the combination of *A*. *tumefaciens* C58C1 harboring p2YN-NbSKη produced NbSKη–nYFP or p2YN-BIN2 produced BIN2-nYFP and *A*. *tumefaciens* C58C1 carrying p2YC-C4 produced C4-cYFP. The BiFC assay was performed as described previously [66]. Emission of the YFP interaction signal was detected using Zeiss LSM780 laser scanning microscope (Carl-Zeiss) at 48 hpi.

### Subcellular fractionation and membrane flotation assays

Subcellular fractionation and immunoblot were conducted as previously described [67–69], with minor modification. 0.5 g of *Agrobacterium*-infiltrated leaves at 2 dpi were gently ground with 1mL of homogenization buffer [50mM Tris-HCl, pH 8.0, 10mM KCl, 3mM MgCl_2,_ 1 mM EDTA, 1mM DTT, 0.1% BSA, 0.3% dextran, and 13% (w/v) sucrose]. Centrifugated at 3,000 *g* for 20 min at 4°C to remove nuclei and large cellular debris and the resulting supernatant was ultracentrifuged at 30,000 *g* for 1h at 4°C to generate the soluble (S30) and the microsomal (P30) fractions. The P30 fraction was re-suspended in the homogenization buffer. All fractions were subjected to SDS-PAGE/western blot analysis.

Membrane flotation assays were performed essentially as described previously [68]. Plant tissues infiltrated at 2 dpi were harvested and ground in homogenization buffer, centrifuged at 3,000 *g* for 20 min at 4°C to discard nuclei and large cellular debris. The resulting supernatant S3 (300 μL) was mixed with 1.6 mL of 85% sucrose (the final concentration of sucrose will be 71.5%), and the mixture was overlaid with 7 mL of 60% sucrose and then 3 mL of 10% sucrose. After centrifugation at 100,000 *g* for 20 h at 4°C. 8–1.5 mL fractions were collected from the top of the tube and subjected to SDS-PAGE/western blot analysis.

### Nuclear-cytoplasmic fractionation assay

Nuclear-cytoplasmic fractionation assays were performed as described previously [70].

### Quantitative RT-PCR (qRT-PCR)

Total RNA was extracted from *N*. *benthamiana* leaf tissues using TRIzol reagent (Invitrogen) according to the manufacturer’s instructions. First strand synthesis was performed using ReverTra Ace qPCR RT Master Mix with gDNA Remover (TOYOBO). Real-time PCR was conducted as described previously [71] using SYBR Green I Master (Roche) according to the manufacturer’s instructions. Primers were used for the amplification of the targets, and the efficiencies of all primers were verified by normal RT-RCR, gel electrophogenesis and melting curve analysis. Primer sequences are listed in Supplemental Table 1. Expression of *actin* gene was used as internal control. The value of each bar indicates the average value of three independent measurements.

### Co-IP assay

*N*. *benthamiana* leaves (0.5g) infiltrated at 2 dpi were harvested and ground in 1 mL IP buffer (50mM Tris-HCl, 150mM NaCl, 10mM MgCl_2_, 5mM DTT, and Triton X-100 0.1%), centrifugated at 8,000 *g* at 4°C for 15 min, then the soluble proteins were immunoprecipitated with 20 μL anti-Myc agarose affinity gel at 4°C for 2 h, the unspecific bound proteins were removed by three consecutive washes with IP buffer with 10 min incubation each at 4°C. The protein complexes were eluted in 200 μL of elution buffer (5mM EDTA, 200mM NH_4_OH) for 20 min. The supernatant and crude extracts were subjected to SDS-PAGE/western blot analysis.

### Production of the construct for *Agrobacterium*-mediated inoculation

The production of constructs for inoculation of TLCYnV has been described previously [11].

### Immunocytochemistry and electron microscopy

Immunocytochemistry assays were performed as described previously [50].

### Cell density

Cell number measurement was performed as described previously [34]. BY-2 suspension cultures at stationary phase were diluted 10-fold in triplicate with fresh MS media containing different hormones, as indicated. Cell density was measured by haemocytometry after maceration [72]. Cell suspension (1 mL) was taken and mixed with an equal volume of 15% (w/v) CrO_3_ and leaf at room temperature overnight. Cells were dispersed by repeatedly passing through a 1 ml disposable pipette tip. Cell number measurements were performed at an interval of 2 days.

### Flow cytometry analysis

Leaves (1 g) for flow cytometry analysis were cut into stripes by sharp blade in 2–3 mL OTTO 1 buffer (100 mM citric acid, 0.5% (v/v) Tween-20, pH 2.0–3.0, stored at 4°C) at 4°C to release the nucleus from leaves, the OTTO 1 buffer containing the nucleus was filtered through 30 μM nylon mesh filter at 4°C into a 1.5 mL tube, centrifuged at 1,000 *g* for 5 min at 4°C, drop off the suspension and resuspended the pellet by 200 μL OTTO 1 buffer at 4°C for 10 min, added 100 μL OTTO2 buffer (400 mM Na_2_HPO_4_^.^12H_2_O pH 2.0–3.0, stored at room temperature) and propidium iodide (PI) staining for 15 min before flow cytometry analysis.

### Phosphorylation *in vitro*

0.5 μg of purified His-NbSKη fusion protein was incubated with 0.5 μg of His-NbCycD1;1 or His tag at room temperature for 30 min in a 20 μL reaction mixture containing 20 mM Tris-HCl (pH 7.5), 10 mM MgCl_2_, 5 mM dithionthreitol, 100 μM ATP, and 4 μl of [γ-^32^P] ATP (3,000 Ci mM). After 30 min, add 4 μL 6×SDS loading buffer, boiled for 30 min, and separated by an 12.5% SDS-PAGE.

### Protein degradation semi-*in vivo*

Protein degradation semi-*in vivo* assays were performed essentially as described previously [73]. A GFP-NbCycD1;1 sample and a GFP sample were harvested from the leaves infiltrated with agrobacterial cultures expressing either GFP-NbCycD1;1 or GFP at 60 hpi. The samples were extracted gently with native extraction buffer (50 mM Tris-HCl, pH 8.0, 0.5 M sucrose, 1 mM MgCl_2_, 10 mM EDTA, 5 mM dithionthreitol). For analysis of NbCycD1;1 degradation, the plant extract harboring GFP-NbCycD1;1 was mixed with chemicals containing 100 mM cycloheximide (CHX), then the extract was split equally into two tubes, one tube was added into ATP (final concentration was 20 mM), the other was add equal volume of extraction buffer. Two tubes were agitated in an Eppendorf Thermomixer at 25°C, equal volume of sample was removed from the tube at different time points. Before agitating, equal volume of sample was removed to tubes for loading control.

For analysis of NbCycD1;1 degradation by 26S proteasome, the plant extract harboring GFP-NbCycD1;1 was mixed with chemicals containing 100 mM cycloheximide (CHX) and 20 mM ATP, then the extract was split equally into two tubes, one tube was added into MG132 (final concentration was 100 μM), the other was add equal volume of DMSO. Two tubes were agitated in an Eppendorf Thermomixer at 25°C, equal volume of sample was removed from the tube at different time points. Before agitating, equal volume of sample was removed to tubes for loading control. Protein levels of GFP-NbCycD1;1 were analyzed with anti-GFP antibody.

## Supporting information

S1 Table Primers used in plasmid construction in this study. (DOCX)

S1 Fig TLCYnV C4 interacts with NbSKη. **(A)** C4 and NbSKη interaction detected in the Y2H assays. Yeast strain Gold co-transformed with the indicated plasmids were subjected to 10-fold serial dilutions, and grown on a SD/-Leu/-Trp/-His/-Ade medium. BD, fused to GAL4 DNA binding domain; AD, fused to GAL4 activation domain. **(B)** BiFC analysis of the interaction between TLCYnV C4 and NbSKη in epidermal cells of H2B-RFP transgenic *N*. *benthamiana* leaves. Scale bar = 50 μm. **(C)** Phylogenetic analysis of the shaggy-related protein kinase η homologues from different species based on the amino acid sequences using Clustal W method from MegAlign software. **(D)** Schematic representation of NbSKη deduced from SMART online software.
(PDF)

S2 Fig BiFC analysis of the interaction between TLCYnV C4 mutants and NbSKη in epidermal cells of H2B-RFP transgenic *N*. *benthamiana* leaves. Scale bar = 50 μm.
(PDF)

S3 Fig The interaction between TLCYnV C4 and NbSKη is necessary for virus infection. Upper panel shows virus DNA accumulation levels in systemic leaves infected by TLCYnV or TLCYnV C4 mutants. Middle panel indicates the total DNA as loading control. Lower panel shows symptoms in plants infected by TLCYnV or TLCYnV C4 mutants.
(PDF)

S4 Fig Western blot analysis shows the nuclear (N) and cytoplasmic (C) distributions of NbSKη protein in *N*. *benthamiana* and *35S*::*C4* transgenic *N*. *benthamiana* plants. PEPC was used as the marker for cytoplasmic fraction, and histone H3 was used as the marker for nuclear fraction. T, N, and C represent total, nuclear and cytoplasmic extracts, respectively.
(PDF)

S5 Fig Identification of the specificity of anti-GSK 3β antibody. (PDF)

S6 Fig TLCYnV C4 mutants not interacting with NbSKη could not change the distribution pattern of NbSKη. **(A)** Subcellular localization of TLCYnV C4 mutants. Confocal micrographs of C4, C4(P32A), C4(P33A), C4(N34A), C4(T35A) or C4(T36A) fused to GFP following agroinfiltration into transgenic *N*. *benthamiana* H2B-RFP marker plants. Scale bar = 50 μm. **(B)** C4-NbSKη interaction compromised mutants do not alter the accumulation level of NbSKη in the nucleus. Confocal micrographs of epidermal cells of H2B-RFP transgenic *N*. *benthamiana* plants co-expressing GFP-NbSKη with C4-CFP, C4(P32A)-CFP C4(P33A)-CFP, C4(N34A)-CFP, C4(T35A)-CFP, or C4(T36A)-CFP. Scale bar = 50 μm. **(C)** Nuclear-cytoplasmic fractionation analysis of the accumulation level of the nuclear-localized NbSKη in wild-type or *N*. *benthamiana* plant tissues infected with PVX, PVX-C4, or PVX-C4 mutants. NbSKη was detected using the rabbit polyclonal antibody raised against GSK3β, PVX was detected using the rabbit polyclonal antibody raised against PVX CP, C4 was detected using the rabbit polyclonal antibody raised against TLCYnV C4, Histone H3 as the nuclear-localized protein marker was detected using the rabbit polyclonal antibody raised against Histone H3, and PEPC as the cytoplasm-localized protein marker was detected using the rabbit polyclonal antibody raised against PEPC. N and C represent nuclear and cytoplasmic extracts, respectively.
(PDF)

S7 Fig Identification of the interaction between TLCYnV C4 and BIN2. **(A)** The interaction between BIN2 and TLCYnV C4 was detected in Y2H assays. Yeast strain Gold co-transformed with indicated plasmids were subjected to 10-fold serial dilutions, and grown on SD/-Leu/-Trp/-His or SD/-Leu/-Trp/-His/-Ade medium. BD, fused to GAL4 DNA binding domain; AD, fused to GAL4 DNA activation domain. **(B)** BiFC analysis of the interaction between BIN2 and TLCYnV C4. Scale bar = 50 μm.
(PDF)

S8 Fig Analysis of the interactions between TLCYnV C4 and BZR1 in Y2H assay. Yeast strain Gold co-transformed with indicated plasmids were subjected to 10-fold serial dilutions, and grown on SD/-Leu/-Trp/-His/-Ade medium. BD, fused to GAL4 DNA binding domain; AD, fused to GAL4 DNA activation domain.
(PDF)

S9 Fig The density of BY-2 suspension cultured in medium containing various hormones. BY-2 cells were diluted and cultured in triplicate in liquid media with various hormones, or without hormone (CK-). Relative density (1.0) represents cell density at 0 day (2.6×10^3^ cells/mL). Error bars show standard error.
(PDF)

S10 Fig The effect of BL or BRZ on the rate of symptom appearance. (PDF)

S11 Fig Subcellular localization of GFP-NbCycD1;1 in the background of H2B-RFP transgenic *N*. *benthamiana* leaves. Scale bar = 50 μm.
(PDF)

S12 Fig NbCycD1;1 plays an important role in TLCYnV C4-induced callus-like tissue formation. **(A)** The phenotype of mock (TRV-GFP) and *NbCycD1;1* silenced (TRV-*NbCycD1;1*) *N*. *benthamiana* plants inoculated with PVX or PVX-C4 at 8 dpi and 21 dpi. Arrows indicate the callus-like tissue. **(B)** Western blot analysis of NbCycD1;1 accumulation level in *N*. *benthamiana* plants under different treatments at 21 dpi. Western blot analysis was conducted with antibodies specific to the indicated proteins.
(PDF)

S13 Fig Southern blot analysis of TLCYnV accumulation level in *N*. *benthamiana* or *NbSKη-*RNAi plants at different time points. (PDF)

S14 Fig The mature leaf phenotype and NbCycD1;1 accumulation level of wild-type, *35S*::*C4*, and *35S*::*NbCycD1;1* transgenic *N*. *benthamiana* plants. **(A)** The mature leaf phenotype of *35S*::*C4* transgenic *N*. *benthamiana* plants mimics that of *35S*::*NbCycD1;1* transgenic *N*. *benthamiana* plants on callus-like tissues formation. **(B)** Western blot analysis of NbCycD1;1 accumulation level in wild-type, *35S*::*C4*, and *35S*::*NbCycD1;1* transgenic *N*. *benthamiana* plants.
(PDF)

S15 Fig NbSKη interacts with NbCycD3;2. **(A-B) Interaction between NbCyclin3;2 and NbSKη was validated in Y2H and BiFC assays.** Scale bar = 50 μm. **(C)** Western blot analysis of the stability of GFP-NbCycD3;2 in wild-type and *35S*::*C4* transgenic *N*. *benthamiana* plants.
(PDF)

S16 Fig Analysis of the interaction between BIN2 and viral proteins *in vivo*. (PDF)

S17 Fig Phenotypes of the wild-type (Col-0) and transgenic *Arabidopsis* plants expressing TYLCCNV βC1, and TLCYnV C4. (PDF)
