# Supplementary material for: Tomato leaf curl Yunnan virus-encoded C4 induces cell division through enhancing stability of Cyclin D 1.1 via impairing NbSKη -mediated phosphorylation in Nicotiana benthamiana
Source: PLoS Pathog. 2018 Jan 2;14(1):e1006789. doi: 10.1371/journal.ppat.1006789 (PMC5766254; doi:10.1371/journal.ppat.1006789)
Supplement: S1 Table — (DOCX) [file ppat.1006789.s001.docx]

**S1 Table Primers used in plasmid construction in this study**

| **Gene** | **Sequence** |
| --- | --- |
| **Cloning primers** |  |
| pGBKT7-TLCTnV C4-F-EcoRI | GAATTCATGGGTCACTGCATCTCCATGTG |
| pGBKT7-TLCTnV C4-R-BamHI | GGATCCGGGCCTCTGCAGCAGCATCAT |
| pGADT7- NbSKη-F-EcoRI | GAATTCATGGCTGATGATAAGGAGATTTCTG |
| pGADT7- NbSKη-R-BamHI | GGATCCCGTCATGTCACCGTGGG |
| p2YC-TLCYnV C4-F-PacI | TTAATTAACATGGGTCACTGCATCTCCATGTG |
| p2YC-TLCYnV C4-R-SpeI | ACTAGTGGGCCTCTGCAGCAGCATCATTAG |
| p2YN-NbSKη-F-AscI | TTAATTAACATGGCTGATGATAAGGAGATTTCTG |
| p2YN-NbSKη-R-SpeI | ACTAGTCGTCATGTCACCGTGGG |
| pGBKT7-TLCTnV C4(27-85aa)-F-EcoRI | GAATTCATGCACACCCTAATACCTCCACCC |
| pGBKT7-TLCTnV C4(44-85aa)-F-EcoRI | GAATTCATGCCAGCTCGGACGTCAAGTCCTAC |
| pGBKT7-TLCTnV C4(1-26+52-85aa)-F | CCAGACATTTCGACATCATCAAGAAGGACGGTGATTAC |
| pGBKT7-TLCTnV C4(1-26+52-85aa)-R | GTAATCACCGTCCTTCTTGATGATGTCGAAATGTCTGG |
| pGBKT7-TLCTnV C4(1-26+62-85aa)-F | CCAGACATTTCGACATCAGTAGTTTTCAGATCGATGGAA |
| pGBKT7-TLCTnV C4(1-26+62-85aa)-R | TTCCATCGATCTGAAAACTACTGATGTCGAAATGTCTGG |
| TLCYnV C4(P32A)-F | ATACCTGCTCCCAATACCACC |
| TLCYnV C4(P32A)-R | GGTGGTATTGGGAGCAGGTAT |
| TLCYnV C4(P33A)-F | ATACCTCCAGCTAATACCACC |
| TLCYnV C4(P33A)-R | GGTGGTATTAGCTGGAGGTAT |
| TLCYnV C4(N34A)-F | CTCCACCCGCTACCACCCAAACTTC |
| TLCYnV C4(N34A)-R | GAAGTTTGGGTGGTAGCGGGTGGAG |
| TLCYnV C4(T35A)-F | CTCCACCCAATGCTACCCAAACTTC |
| TLCYnV C4(T35A)-R | GAAGTTTGGGTAGCATTGGGTGGAG |
| TLCYnV C4(T36A)-F | CTCCACCCAATACCGCTCAAACTTC |
| TLCYnV C4(T36A)-R | GAAGTTTGAGCGGTATTGGGTGGAG |
| PVX-TLCYnV C4-F-AscI | GGCGCGCCCATGGGTCACTGCATCTCCATGTG |
| PVX-TLCYnV C4-R-SalI | GTCGACTTAGGGCCTCTGCAGCATC |
| pCHF3-TLCYnV C4-GFP/CFP/  Myc-F-KpnI | GGTACCATGGGTCACTGCATCTCCATGTG |
| pCHF3-TLCYnV C4-GFP/CFP  /Myc-R-BamHI | GGATCCGGGCCTCTGCAGCAGCATCATTAG |
| pGD-GFP- NbSKη-F-SalI | GTCGACATGGCTGATGATAAGGAGATTTCTG |
| pGD-GFP- NbSKη-R-BamHI | GGATCCCGTCATGTCACCGTGGG |
| TRV- NbSKη-F-BamHI | GGATCCAATGGTGCAGTCACTGGTCATATAATTTC |
| TRV- NbSKη-R-XhoI | CTCGAGCCACAGTCTCCCCGTTTTCCAG |
| TRV-NbCyclin D1;1-F-BamHI | GGATCCGCCGGTCTTATTGAAGACGAACG |
| TRV-NbCyclin D1;1-R-XhoI | CTCGAGGAAAGCTAGCTTCTTGAATATTTG |
| pGD-GFP- NbCycD1;1-F-SalI | GTCGACATGTCAGTCTCGTGCTCCG |
| pGD-GFP- NbCycD1;1-R-BamHI | GGATCCTTAACAGGAACTTGCTCTGTCG |
| pGADT7-NbCycD3;2-F-EcoRI | GAATTCATGGCAATAGAACAAAATGA |
| pGADT7-NbCycD3;2-R-BamHI | GGATCCTTAGGTGATTGATTTTAAATGAG |
| p2YN/p2YC--NbCycD3;2-F-PacI | TTAATTAAGATGGCAATAGAACAAAATGA |
| p2YN/p2YC--NbCycD3;2-R-SpeI | ACTAGTGGTGATTGATTTTAAATGAG |
| pGBKT7- NbCycD1;1-F-EcoRI | GAATTCATGTCAGTCTCGTGCTCCG |
| pGBKT7- NbCycD1;1-R-  BamHI | GGATCCTTAACAGGAACTTGCTCTGTCG |
| p2YC- NbCycD1;1-F-PacI | TTAATTAACATGTCAGTCTCGTGCTCCG |
| p2YC- NbCycD1;1-R-SpeI | ACTAGTACAGGAACTTGCTCTGTCG |
| pCambia-Flag- NbSKη-F | GGTACCATGGCTGATGATAAGGAGATTTCTG |
| pCambia-Flag- NbSKη-R | GGATCCTTACGTCATGTCACCGTGGG |
| pGex4T-3- NbCycD1;1-F-BamHI | GGATCCATGTCAGTCTCGTGCTCCG |
| pGex4T-3- NbCycD1;1-R-SalI | GTCGACACAGGAACTTGCTCTGTCG |
| pET-32a- NbCycD1;1-F-BamHI | GGATCCATGTCAGTCTCGTGCTCCG |
| pET-32a- NbCycD1;1-R-EcoRI | GAATTCACAGGAACTTGCTCTGTCG |
| pET-32a- NbSKη-F-BamHI | GGATCC ATGGCTGATGATAAGGAGATTTCTG |
| pET-32a- NbSKη-SalI | GTCGAC TTACGTCATGTCACCGTGGG |
| pET-32a- NbCycD1;1(T328A)-R-  EcoRI | GAATTCTTAACAGGAACTTGCTCTGTCGTCAACTGCATTCCACCAGCT |
| pGD-GFP- NbCycD1;1(T328A)-R-  BamHI | GGATCCTTAACAGGAACTTGCTCTGTCGTCAACTGCATTCCACCAGCT |
| pCHF3- NbCycD1;1-F-KpnI | GGTACCATGTCAGTCTCGTGCTCCG |
| pCHF3- NbCycD1;1-R-BamHI | GGATCCTTAACAGGAACTTGCTCTGTCG |
| pGADT7-BIN2-F-EcoRI | GAATTCATGGCTGATGATAAGGAGATGCCTG |
| pGADT7-BIN2-R-BamHI | GGATCCTTAAGTTCCAGATTGATTCAAGAA |
| p2YN-BIN2-F-PacI | TTAATTAAC ATGGCTGATGATAAGGAGATGCCTG |
| p2YN-BIN2-R-SpeI | ACTAGTAGTTCCAGATTGATTCAAGAA |
| pGBKT7-BZR1-F-EcoRI | GAATTCATGACTTCGGATGGAGCTACGTCG |
| pGBKT7-BZR1-R-BamHI | GGATCCTCAACCACGAGCCTTCCCATTTC |
| p2YC-BZR1-F-PacI | TTAATTAAC ATGACTTCGGATGGAGCTACGTCG |
| p2YC-BZR1-R-SpeI | ACTAGTACCACGAGCCTTCCCATTT |
| pCHF3-Flag-Myc-TYLCV V2-F | GGATCCATGTCGAAGCGACCAGGCGATAT |
| pCHF3-Flag-Myc-TYLCV V2-R | GTCGACTTAATTTGATATTGAATCATAGAAATAGATGCGTATTTTC |
| pCHF3-Flag-Myc-TYLCCNV-βC1-F | GGATCCATGACTATCAAATACAACAACATGAAGG |
| pCHF3-Flag-Myc-TYLCCNV βC1-R | GTCGACTCATACATCTGAATTCATAAATACATCATATTC |
| pCHF3-Flag-Myc-CaLCuV AL2 -F | GGATCCATGCAAAATTCATCACTCTTGAAG |
| pCHF3-Flag-Myc-CaLCuV AL2 -R | GTCGACCTACTTAAATATGTCGGCCCAGAAG |
| **Quantitative-PCR primer pairs** |  |
| qPCR-NbSKη-F | AATGGTGCTGTCACTGGTCACATAATTT |
| qPCR-NbSKη-R | CATAGATGAGTGGCATCCTTTGGTT |
| qPCR-Nbactin-F | CAATCCAGACACTGTACTTTCTCTC |
| qPCR-Nbactin-R | AAGCTGCAGGTATCCATGAGACTA |
| qPCR-Atactin-F | GATTTGGCATCACACTTTCTACAATG |
| qPCR-Atactin-R | GTTCCACCACTGAGCACAATG |
| qPCR-AtDWF4-F | CATAAAGCTCTTCAGTCACGAGCAAC |
| qPCR-AtDWF4-R | CATCGTCTGTTCTTTGTTTCCTAACATG |
| qPCR-AtCPD-F | TTGCTCAACTCAAGGAAGAGCATG |
| qPCR-AtCPD-R | CGATGATGTTAGCCACTCGTAGC |
