# Supplementary figures and images for: Tomato leaf curl Yunnan virus-encoded C4 induces cell division through enhancing stability of Cyclin D 1.1 via impairing NbSKη -mediated phosphorylation in Nicotiana benthamiana
Source: PLoS Pathog. 2018 Jan 2;14(1):e1006789. doi: 10.1371/journal.ppat.1006789 (PMC5766254; doi:10.1371/journal.ppat.1006789)

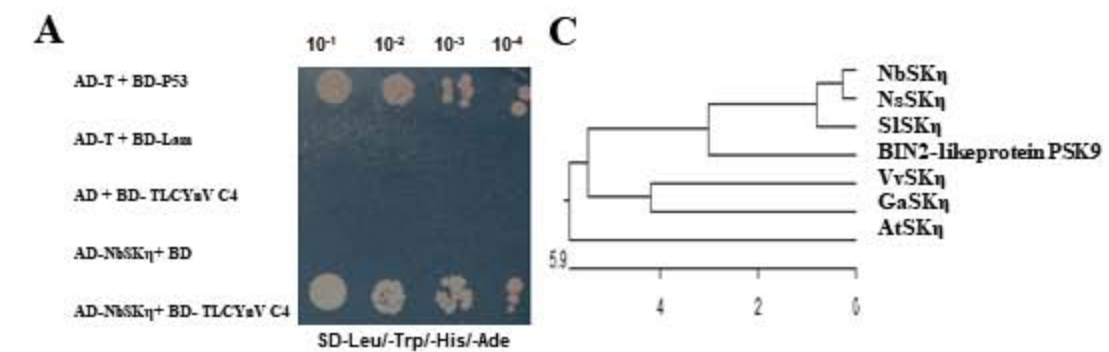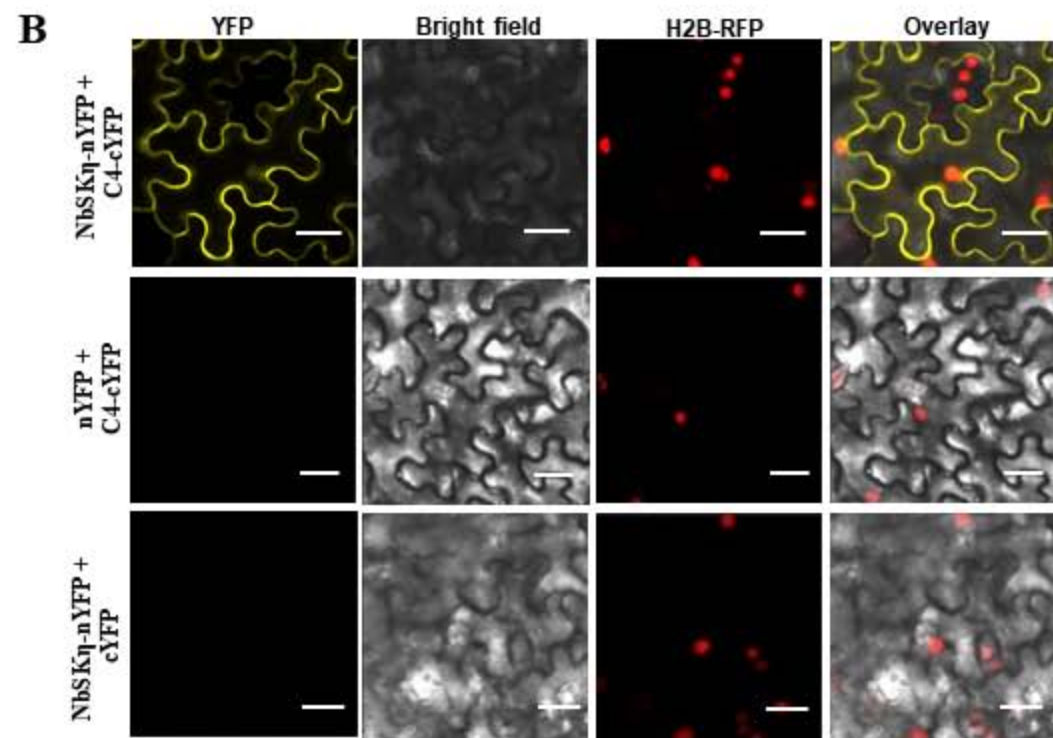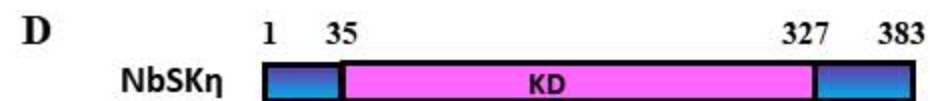

Supplement: S1 Fig — (A) C4 and NbSKη interaction detected in the Y2H assays. Yeast strain Gold co-transformed with the indicated plasmids were subjected to 10-fold serial dilutions, and grown on a SD/-Leu/-Trp/-His/-Ade medium. BD, fused to GAL4 DNA binding domain; AD, fused to GAL4 activation domain. (B) BiFC analysis of the interaction between TLCYnV C4 and NbSKη in epidermal cells of H2B-RFP transgenic N. benthamiana leaves. Scale bar = 50 μm. (C) Phylogenetic analysis of the shaggy-related protein kinase η homologues from different species based on the amino acid sequences using Clustal W method from MegAlign software. (D) Schematic representation of NbSKη deduced from SMART online software. (PDF) [file ppat.1006789.s002.pdf]

# NbSK $\eta$ -nYFP+

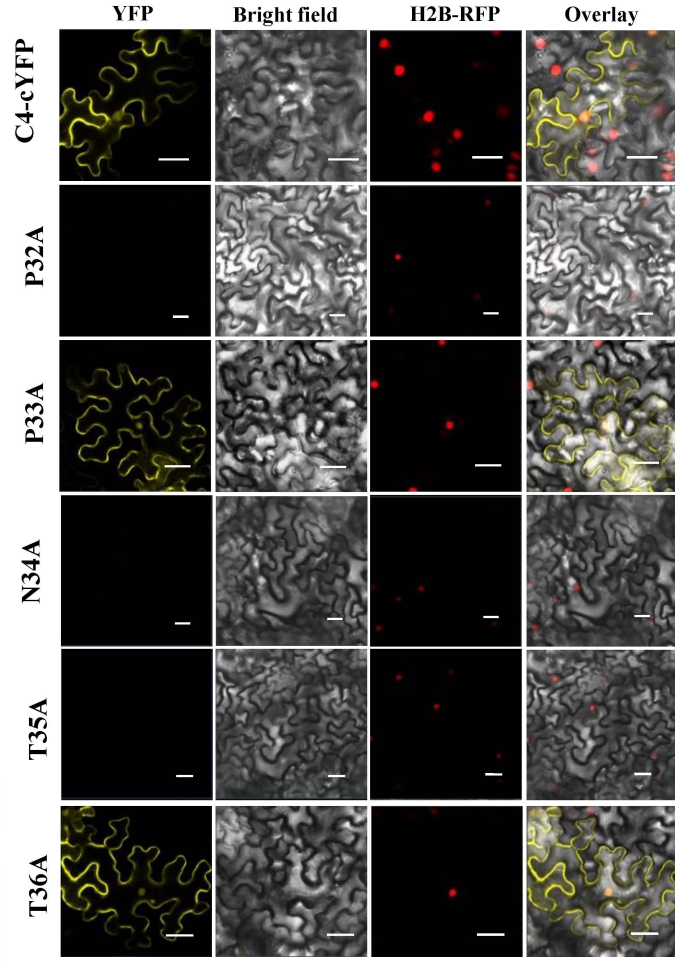

Supplement: S2 Fig — Scale bar = 50 μm. (PDF) [file ppat.1006789.s003.pdf]

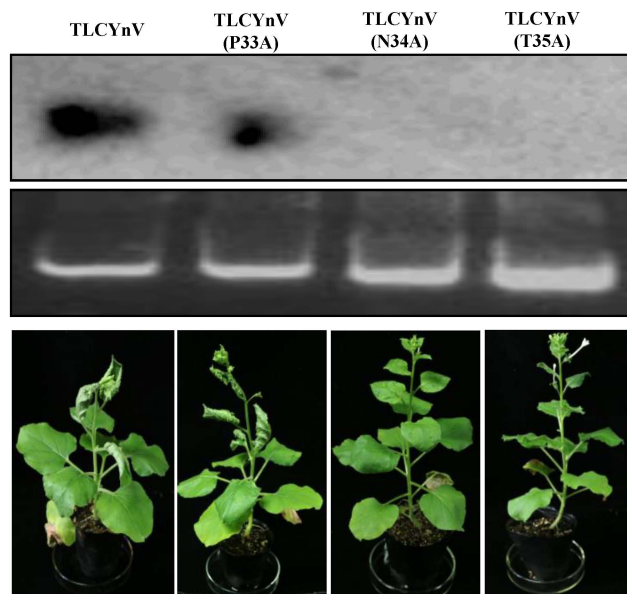

Supplement: S3 Fig — Upper panel shows virus DNA accumulation levels in systemic leaves infected by TLCYnV or TLCYnV C4 mutants. Middle panel indicates the total DNA as loading control. Lower panel shows symptoms in plants infected by TLCYnV or TLCYnV C4 mutants. (PDF) [file ppat.1006789.s004.pdf]

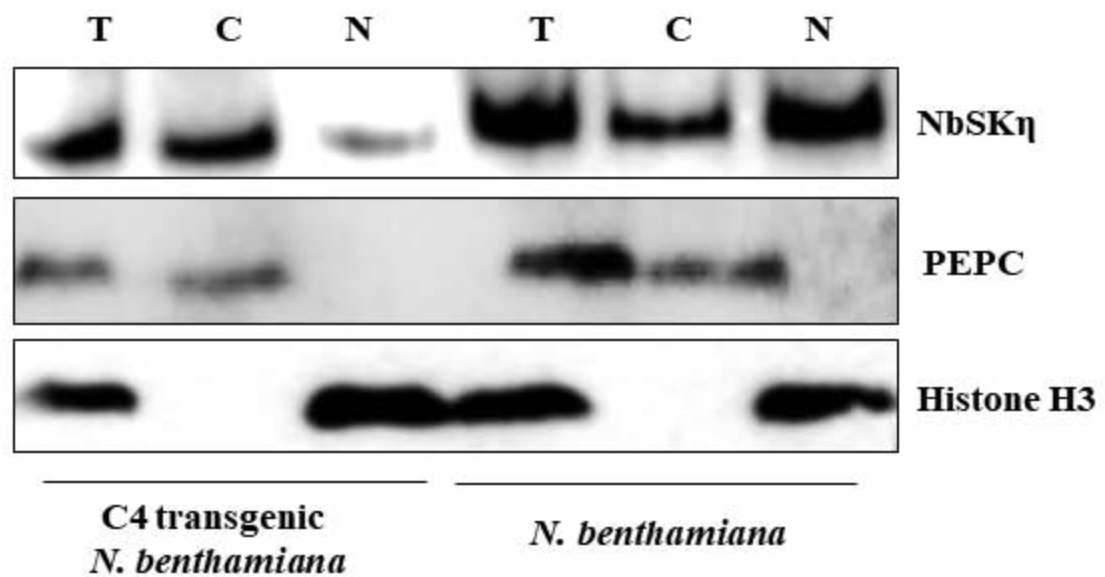

Supplement: S4 Fig — PEPC was used as the marker for cytoplasmic fraction, and histone H3 was used as the marker for nuclear fraction. T, N, and C represent total, nuclear and cytoplasmic extracts, respectively. (PDF) [file ppat.1006789.s005.pdf]

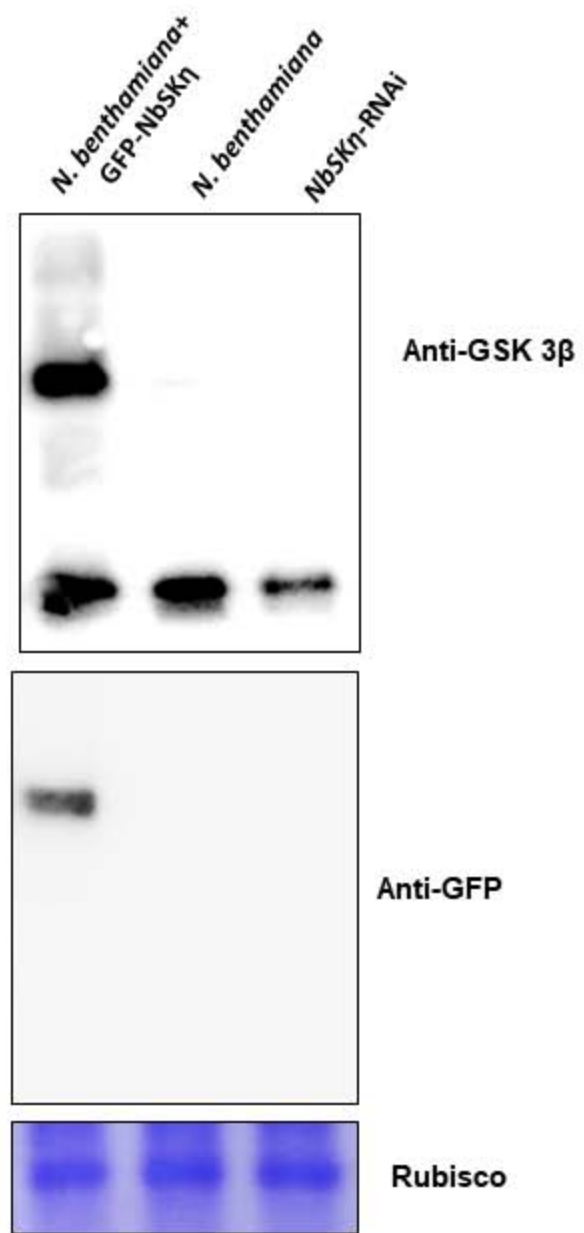

Supplement: S5 Fig — (PDF) [file ppat.1006789.s006.pdf]

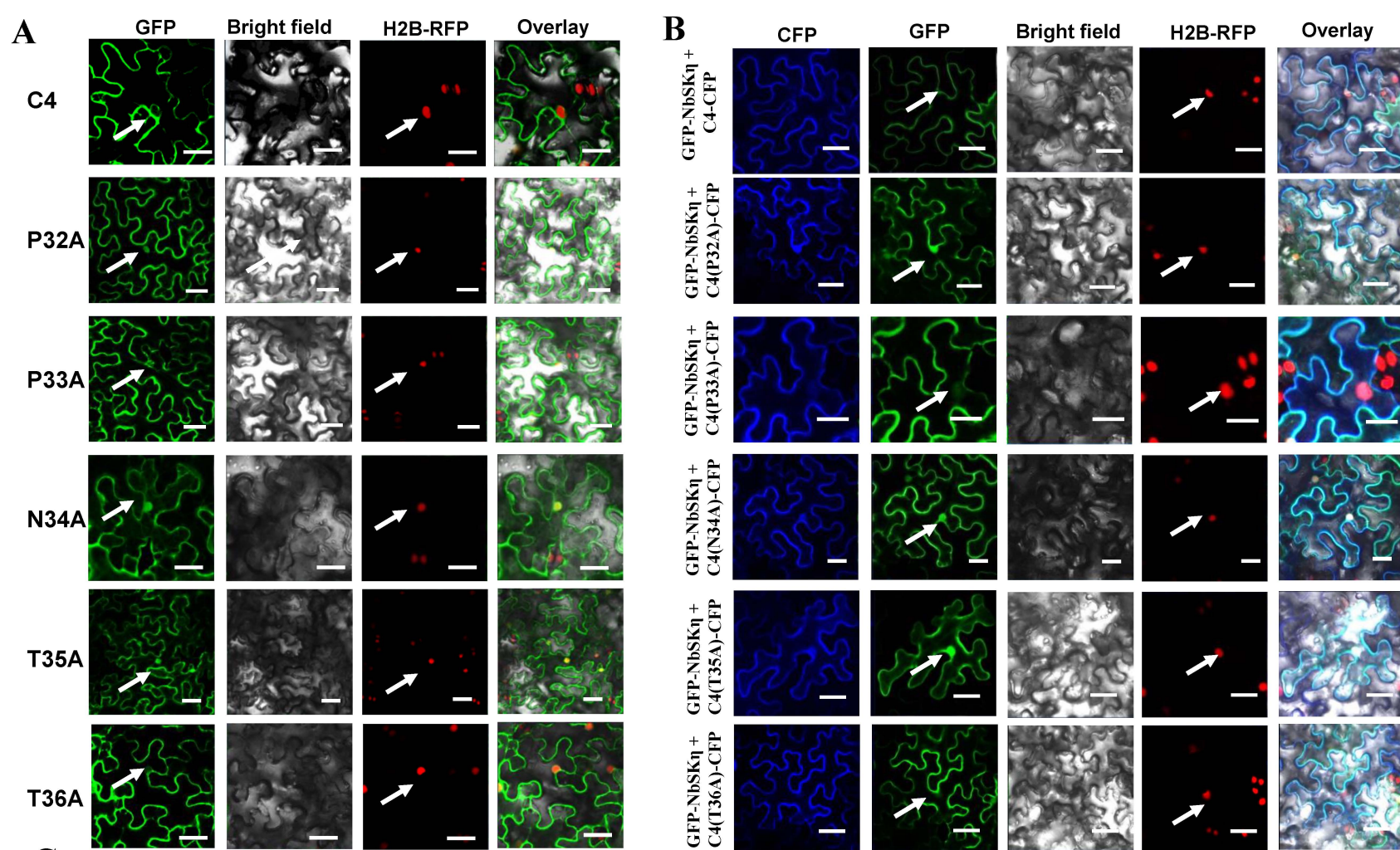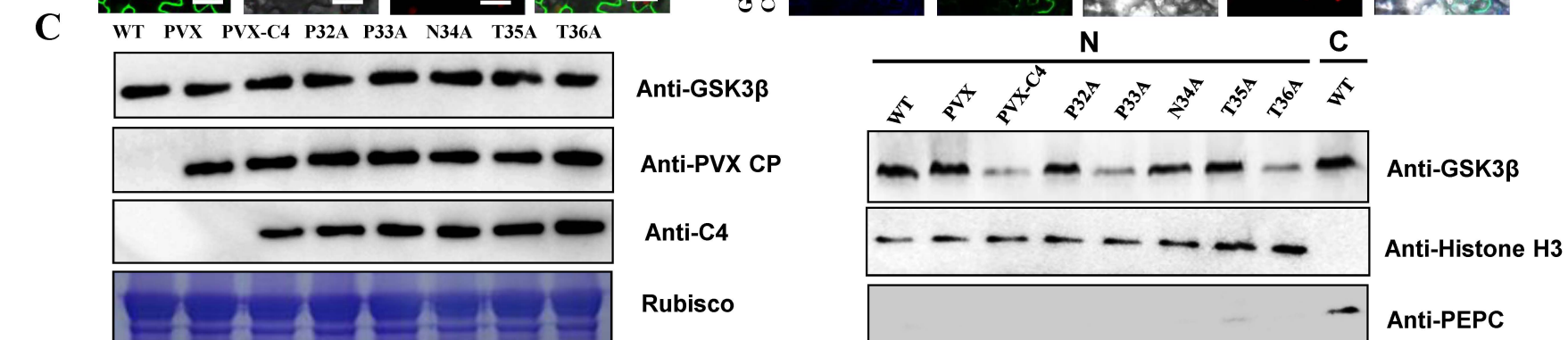

Supplement: S6 Fig — (A) Subcellular localization of TLCYnV C4 mutants. Confocal micrographs of C4, C4(P32A), C4(P33A), C4(N34A), C4(T35A) or C4(T36A) fused to GFP following agroinfiltration into transgenic N. benthamiana H2B-RFP marker plants. Scale bar = 50 μm. (B) C4-NbSKη interaction compromised mutants do not alter the accumulation level of NbSKη in the nucleus. Confocal micrographs of epidermal cells of H2B-RFP transgenic N. benthamiana plants co-expressing GFP-NbSKη with C4-CFP, C4(P32A)-CFP C4(P33A)-CFP, C4(N34A)-CFP, C4(T35A)-CFP, or C4(T36A)-CFP. Scale bar = 50 μm. (C) Nuclear-cytoplasmic fractionation analysis of the accumulation level of the nuclear-localized NbSKη in wild-type or N. benthamiana plant tissues infected with PVX, PVX-C4, or PVX-C4 mutants. NbSKη was detected using the rabbit polyclonal antibody raised against GSK3β, PVX was detected using the rabbit polyclonal antibody raised against PVX CP, C4 was detected using the rabbit polyclonal antibody raised against TLCYnV C4, Histone H3 as the nuclear-localized protein marker was detected using the rabbit polyclonal antibody raised against Histone H3, and PEPC as the cytoplasm-localized protein marker was detected using the rabbit polyclonal antibody raised against PEPC. N and C represent nuclear and cytoplasmic extracts, respectively. (PDF) [file ppat.1006789.s007.pdf]

**AD-BZR1 + BD-C4**

**AD-BIN2 + BD-C4**

**AD-BIN2 + BD-BZR1**

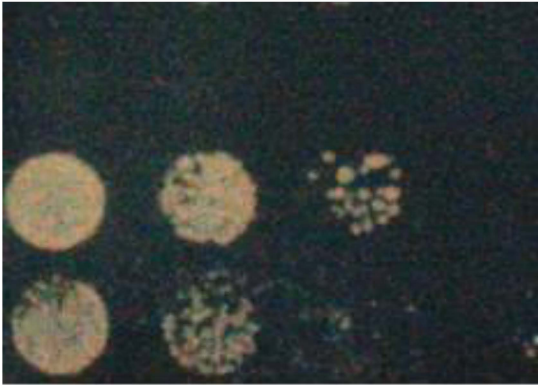

**SD-Leu-Trp-His-Ade**

Supplement: S8 Fig — Yeast strain Gold co-transformed with indicated plasmids were subjected to 10-fold serial dilutions, and grown on SD/-Leu/-Trp/-His/-Ade medium. BD, fused to GAL4 DNA binding domain; AD, fused to GAL4 DNA activation domain. (PDF) [file ppat.1006789.s009.pdf]

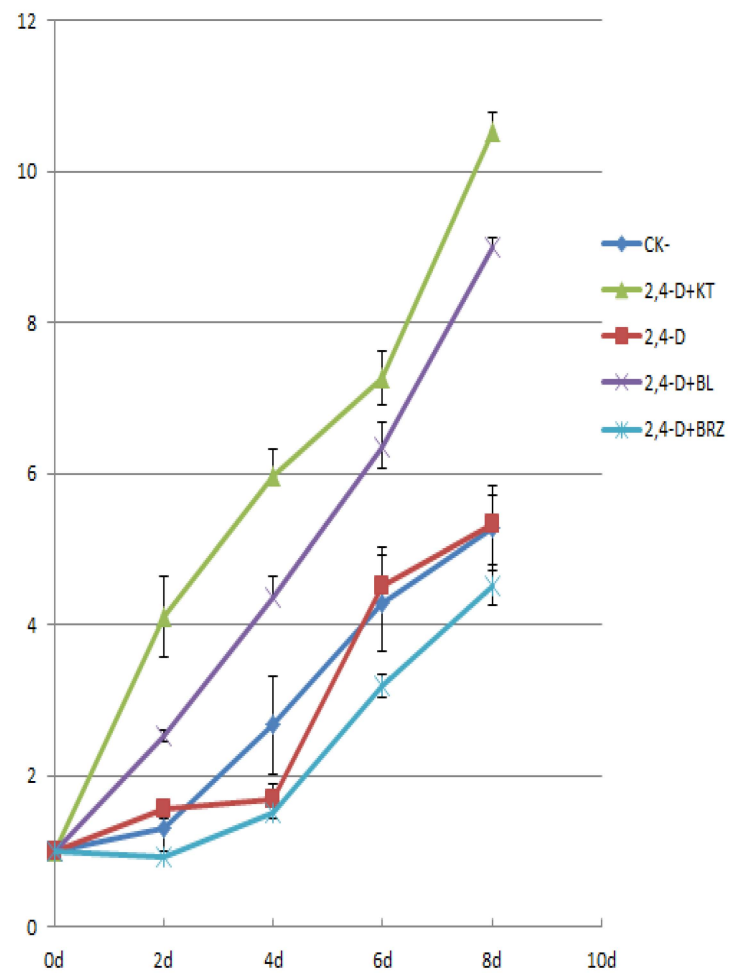

Supplement: S9 Fig — BY-2 cells were diluted and cultured in triplicate in liquid media with various hormones, or without hormone (CK-). Relative density (1.0) represents cell density at 0 day (2.6×10^3^ cells/mL). Error bars show standard error. (PDF) [file ppat.1006789.s010.pdf]

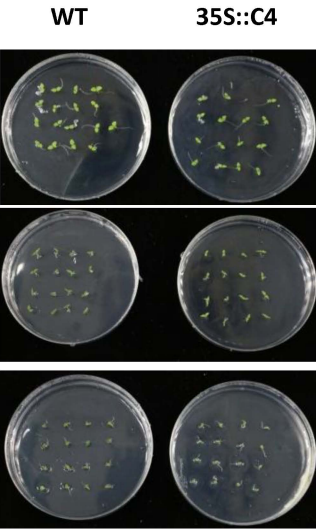

MS

MS+Brassinolide(BL)

MS+Brassinazole(Brz)

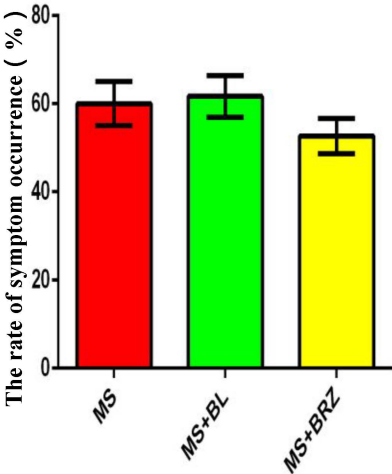

Supplement: S10 Fig — (PDF) [file ppat.1006789.s011.pdf]

GFP-NbCycD1;1

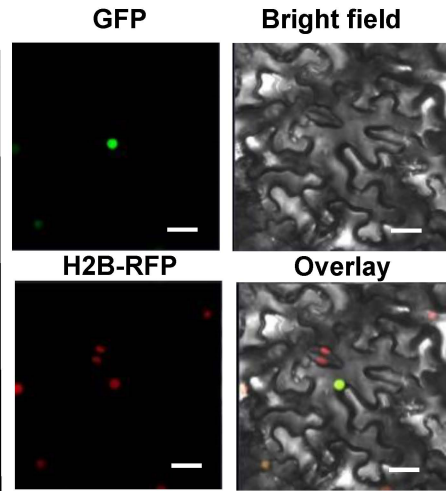

Supplement: S11 Fig — Scale bar = 50 μm. (PDF) [file ppat.1006789.s012.pdf]

*NbSK7*-RNAi

*N. benthamiana*

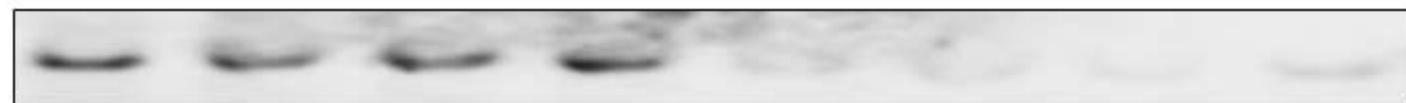

10 dpi

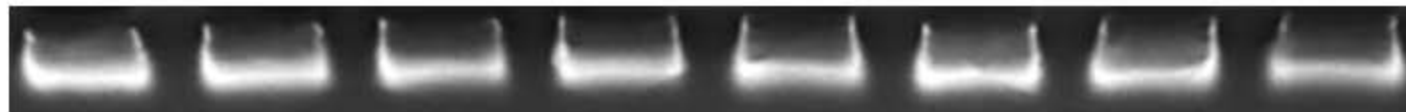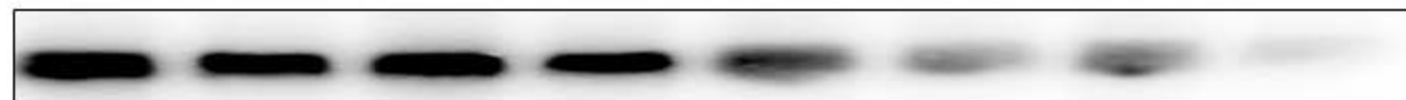

35 dpi

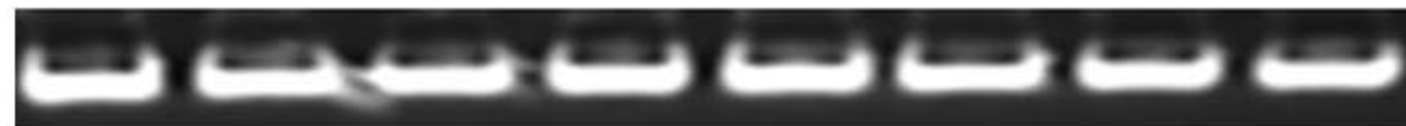

Supplement: S13 Fig — (PDF) [file ppat.1006789.s014.pdf]

A

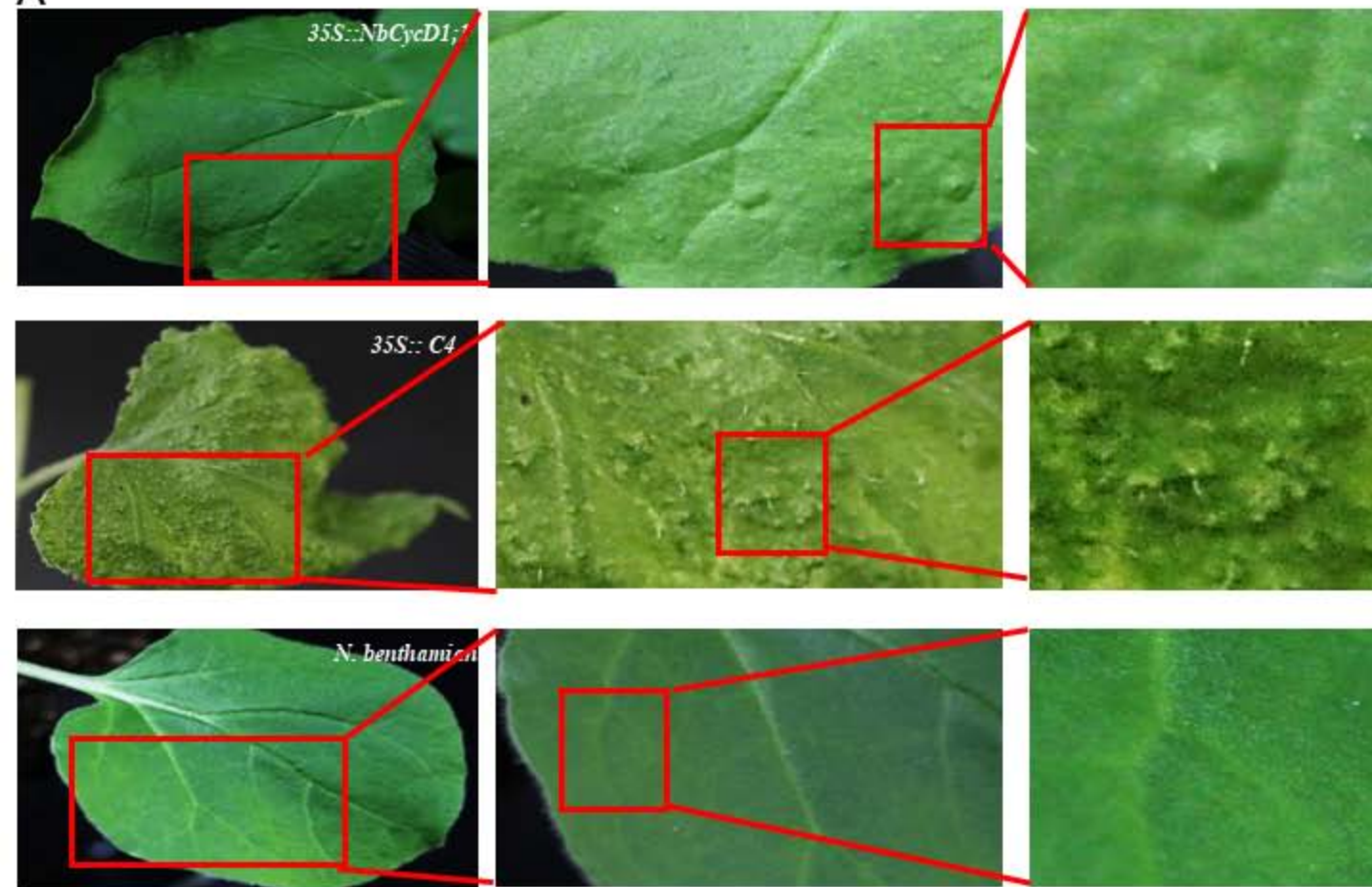

B

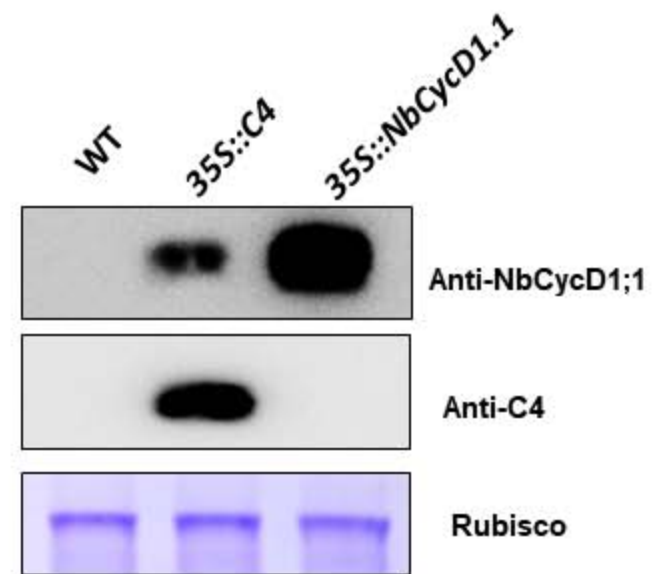

Supplement: S14 Fig — (A) The mature leaf phenotype of 35S::C4 transgenic N. benthamiana plants mimics that of 35S::NbCycD1;1 transgenic N. benthamiana plants on callus-like tissues formation. (B) Western blot analysis of NbCycD1;1 accumulation level in wild-type, 35S::C4, and 35S::NbCycD1;1 transgenic N. benthamiana plants. (PDF) [file ppat.1006789.s015.pdf]

A

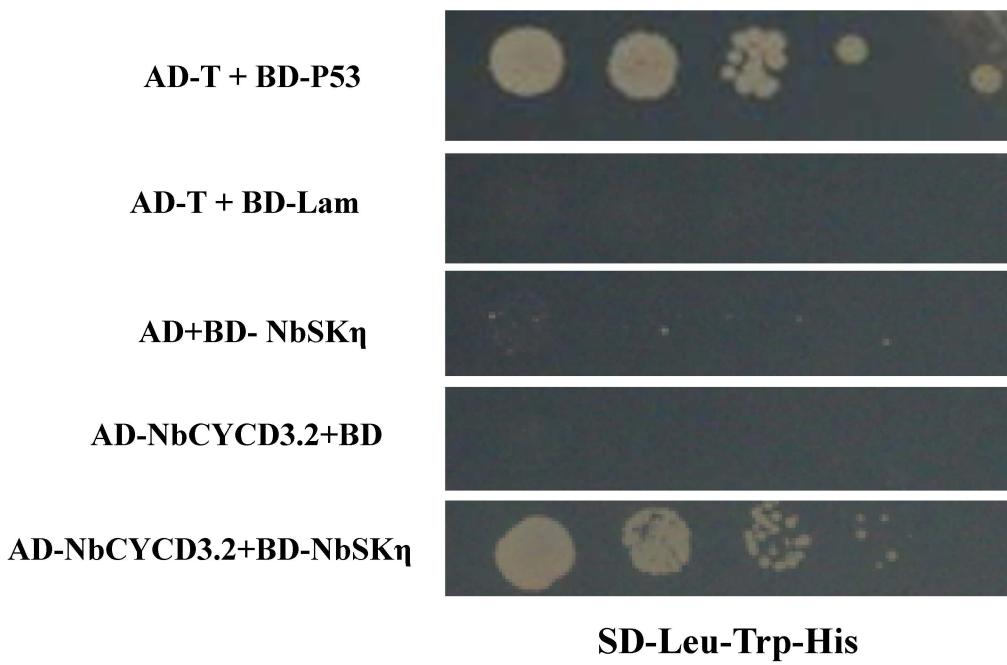

B

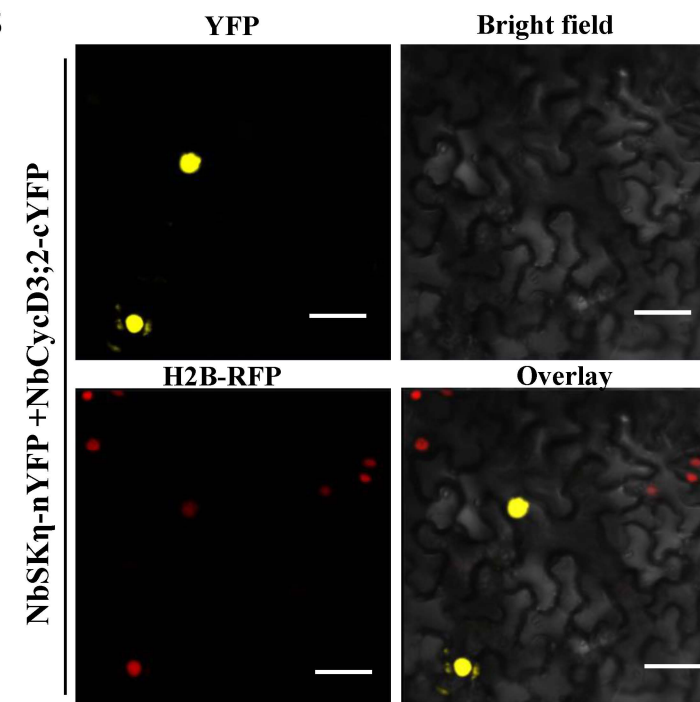

C

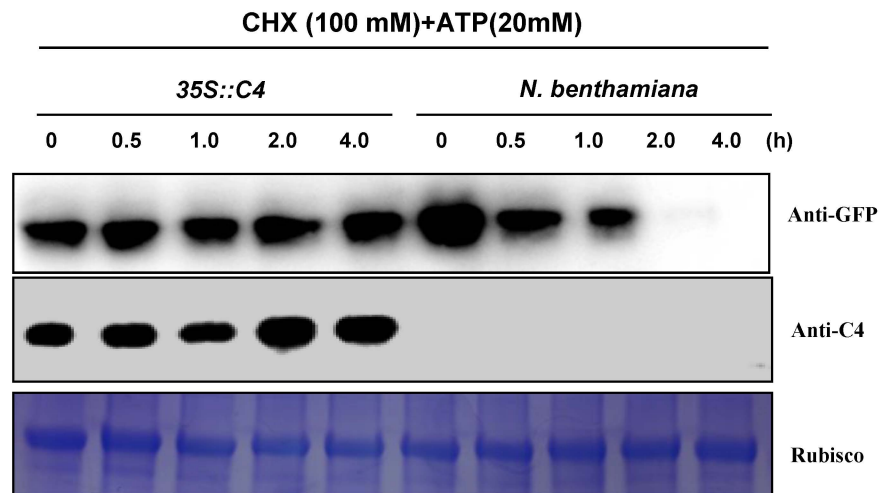

Supplement: S15 Fig — (A-B) Interaction between NbCyclin3;2 and NbSKη was validated in Y2H and BiFC assays. Scale bar = 50 μm. (C) Western blot analysis of the stability of GFP-NbCycD3;2 in wild-type and 35S::C4 transgenic N. benthamiana plants. (PDF) [file ppat.1006789.s016.pdf]

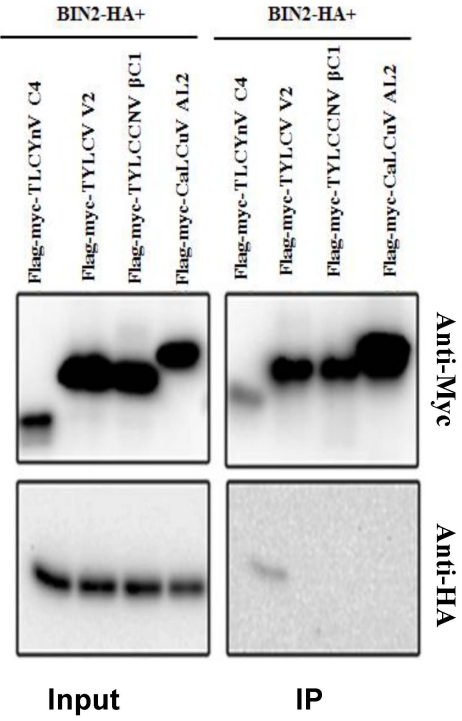

Supplement: S16 Fig — (PDF) [file ppat.1006789.s017.pdf]

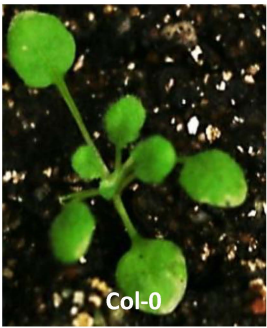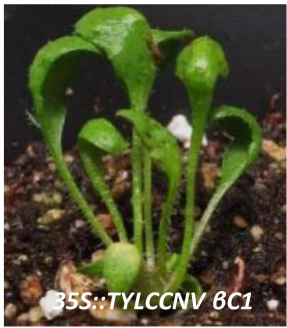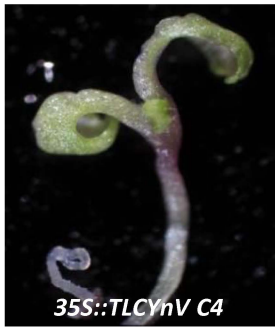

Supplement: S17 Fig — (PDF) [file ppat.1006789.s018.pdf]
