# Supplementary material for: Tomato leaf curl Yunnan virus-encoded C4 induces cell division through enhancing stability of Cyclin D 1.1 via impairing NbSKη -mediated phosphorylation in Nicotiana benthamiana
Source: PLoS Pathog. 2018 Jan 2;14(1):e1006789. doi: 10.1371/journal.ppat.1006789 (PMC5766254; doi:10.1371/journal.ppat.1006789)
Supplement: S7 Fig — (A) The interaction between BIN2 and TLCYnV C4 was detected in Y2H assays. Yeast strain Gold co-transformed with indicated plasmids were subjected to 10-fold serial dilutions, and grown on SD/-Leu/-Trp/-His or SD/-Leu/-Trp/-His/-Ade medium. BD, fused to GAL4 DNA binding domain; AD, fused to GAL4 DNA activation domain. (B) BiFC analysis of the interaction between BIN2 and TLCYnV C4. Scale bar = 50 μm. (PDF) [file ppat.1006789.s008.pdf]

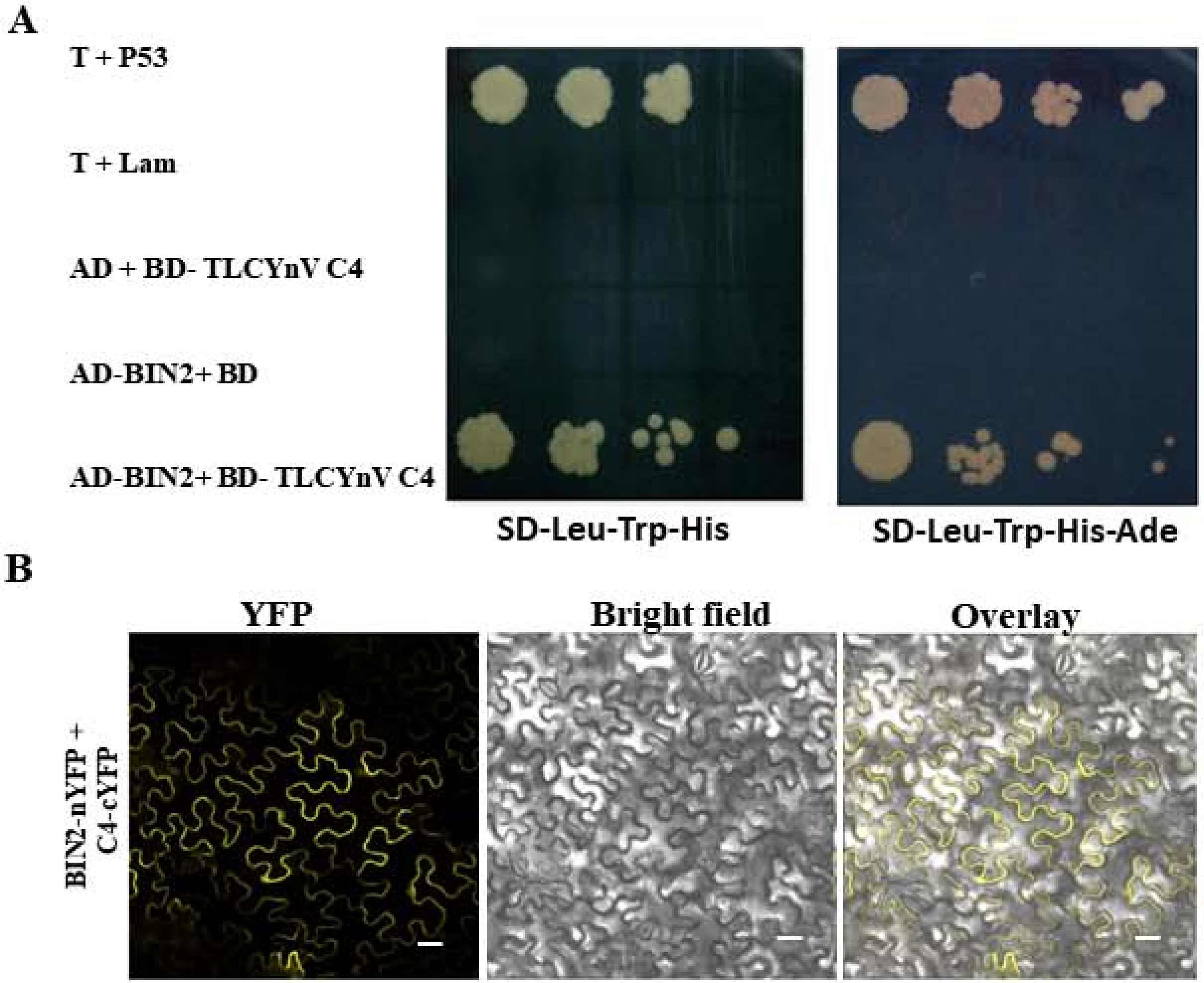
A
T + P53
T + Lam
AD + BD- TLCYnV C4
AD-BIN2+ BD
AD-BIN2+ BD- TLCYnV C4
SD-Leu-Trp-His
SD-Leu-Trp-His-Ade
B
YFP
Bright field
Overlay
BIN2-nYFP +
C4-cYFP
