# Supplementary material for: Tomato leaf curl Yunnan virus-encoded C4 induces cell division through enhancing stability of Cyclin D 1.1 via impairing NbSKη -mediated phosphorylation in Nicotiana benthamiana
Source: PLoS Pathog. 2018 Jan 2;14(1):e1006789. doi: 10.1371/journal.ppat.1006789 (PMC5766254; doi:10.1371/journal.ppat.1006789)
Supplement: S12 Fig — (A) The phenotype of mock (TRV-GFP) and NbCycD1;1 silenced (TRV-NbCycD1;1) N. benthamiana plants inoculated with PVX or PVX-C4 at 8 dpi and 21 dpi. Arrows indicate the callus-like tissue. (B) Western blot analysis of NbCycD1;1 accumulation level in N. benthamiana plants under different treatments at 21 dpi. Western blot analysis was conducted with antibodies specific to the indicated proteins. (PDF) [file ppat.1006789.s013.pdf]

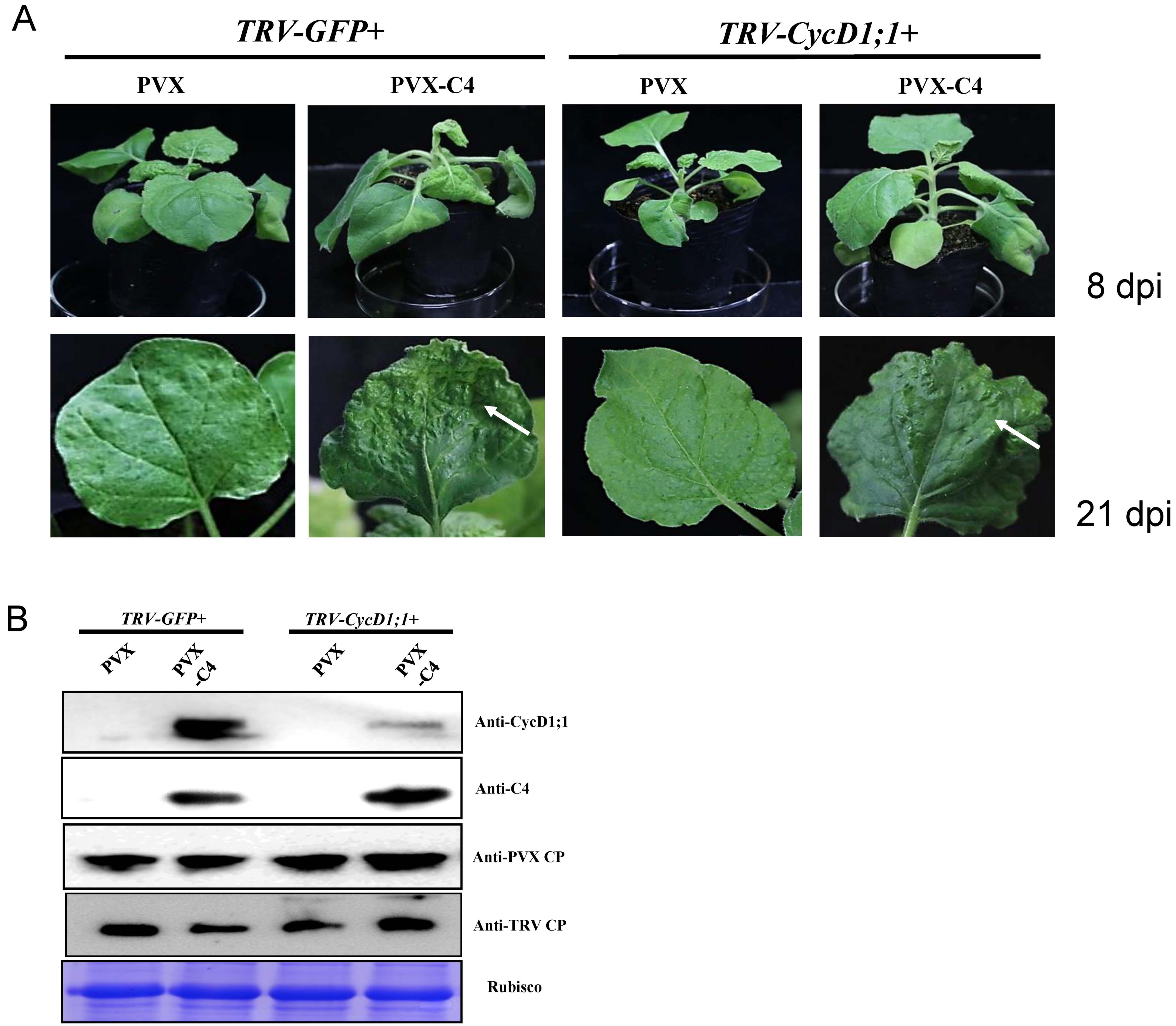
A
TRV-GFP+
TRV-CycD1;1+
PVX
PVX-C4
PVX
PVX-C4
8 dpi
21 dpi
B
TRV-GFP+
TRV-CycD1;1+
PVX
PVX-C4
PVX
PVX-C4
Anti-CycD1;1
Anti-C4
Anti-PVX CP
Anti-TRV CP
Rubisco
